# Long non-coding RNA H19 promotes colorectal cancer metastasis via binding to hnRNPA2B1

**DOI:** 10.1186/s13046-020-01619-6

**Published:** 2020-07-23

**Authors:** Yuhui Zhang, Weibin Huang, Yujie Yuan, Jin Li, Jing Wu, Jie Yu, Yulong He, Zhewei Wei, Changhua Zhang

**Affiliations:** 1 Department of Gastrointestinal Surgery, the First Affiliated Hospital of Sun Yat-sen University, 58 Zhongshan 2nd Road, Guangzhou, 510080 Guangdong China; 2 Center for Digestive Disease, the Seventh Affiliated Hospital of Sun Yat-sen University, 628 Zhenyuan Road, Shenzhen, 518000 Guangdong China

**Keywords:** Colorectal cancer, Metastasis, H19, hnRNPA2B1, EMT

## Abstract

**Background:**

Long non-coding RNA H19 was demonstrated to be significantly correlated with tumor metastasis. However, the specific functions of H19 in colorectal cancer (CRC) metastasis and the underlying mechanism are still largely unclear.

**Methods:**

Use public database to screen the potential lncRNA crucial for metastasis in colorectal cancer. The expression of H19 in clinical CRC specimens was detected by qRT-PCR. The effect of H19 on the metastasis of CRC cells was investigated by transwell, wound healing assays, CCK-8 assays and animal studies. The potential proteins binding to H19 were identified by LC-MS and verified by RNA immunoprecipitation (RIP). The expression of indicated RNA and proteins were measured by qRT-PCR or western blot.

**Results:**

We found the expression of lncRNA H19 was significantly upregulated in primary tumor and metastatic tissues, correlated with poor prognosis in CRC. Ectopic H19 expression promoted the metastasis of colorectal cancer cells in vitro and in vivo, and induced epithelial-to-mesenchymal transition (EMT). Mechanistically, H19 directly bound to hnRNPA2B1. Knockdown of hnRNPA2B1 attenuated the H19-induce migration and invasion in CRC cells. Furthermore, H19 stabilized and upregulated the expression of Raf-1 by facilitated the interaction between hnRNPA2B1 and Raf-1 mRNA, resulting in activation of Raf-ERK signaling.

**Conclusions:**

Our findings demonstrate the role of H19/hnRNPA2B1/EMT axis in regulation CRC metastasis, suggested H19 could be a potential biomarker to predict prognosis as well as a therapeutic strategy for CRC.

## Background

Colorectal cancer (CRC) is the third most prevalent malignancies, accounts for 881,000 deaths around the world in 2018 [1]. Metastasis is common in CRC, which is the major cause for CRC associated deaths [2]. The 5-year survival rate for patients with unresectable metastases was under 10% in CRC, while for stage I disease was 90% [3]. Therefore, to understand the molecular mechanisms driving metastasis and improve treatments strategy for metastasis are of urgent need in CRC.

Tumor metastasis is a multi-step process comprising of a wide variety of molecular events [4]. Increasing long non-coding RNAs (lncRNA) have recently emerged as promising mechanism for metastases in various kinds of cancers [5]. LncRNA comprise of a series of transcripts more than 200 nt in length and occupy limited capacity to translate into proteins [6]. Recent findings have revealed that almost 98% of human transcriptome are non-coding RNAs [7], suggesting lncRNAs may involve in a wide range of pathophysiological processes [8, 9]. Although several lncRNAs have been proved to be of great importance in the dysregulation of proliferation, apoptosis, migration, invasion and chemoresistance for CRC [10–13], the role of most other lncRNAs in colorectal cancer are rarely studied. Therefore, to identify potential lncRNAs involving metastases in CRC is of great need. By analyzing the public database, H19 was one of most overexpressed lncRNAs in primary tumor and metastatic tissues compared with adjacent normal tissues in CRC. Aberrant expression of H19 has been demonstrated in various kinds of malignancies including lung cancer, gastric cancer and pancreatic cancer [14–16]. Furthermore, Zhou et al. reported that H19 lead to epithelial-to-mesenchymal transition (EMT), a pivotal step in cancer metastasis [17], by acting as miRNA sponges to inhibit the functions of related miRNA [18]. During EMT, epithelial cells acquire mesenchymal features, beneficial for migration and invasion of cancer cells, and eventually promotes infiltration and metastasis [19]. Recent studies have revealed lncRNAs trigger EMT and subsequently lead to tumor metastasis [20, 21]. Nevertheless, the exact effect of H19 in CRC and the mechanism in regulating metastasis is remain largely unknown.

In the current study, we identified lncRNA H19 as one of the lncRNAs increased most substantially in primary colorectal cancer tissues, which was further upregulated in metastatic tissues by analyzing public dataset and confirmed with clinical specimens. Functional analyses demonstrated that H19 promotes the metastases of colorectal cancer cells both in vitro and in vivo. Furthermore, mechanistic studies demonstrated that by directly binding to heterogeneous nuclear ribonucleoprotein A2B1 (hnRNPA2B1), H19 activate Raf-ERK signaling and induce EMT, resulting in the metastases of CRC cells. Collectively, we suggested that H19 could be a potential prognosis biomarker and therapeutic target for colorectal cancer.

## Materials and methods

### Patient and clinical samples

A total of 60 pairs of colorectal cancer samples and corresponding adjacent non-tumor colonic epithelium tissues, as well as 11 liver metastasis specimens were acquired from patients at The First Affiliated Hospital, Sun Yat-sen University (Guangzhou, China). Specimens were frozen in liquid nitrogen immediately after resection and stored at − 80 °C. These tissue samples were examined by pathologists. All patients provided written informed consent and the Ethics Committee of The First Affiliated Hospital, Sun Yat-sen University approved the current study.

### Cell lines and culture conditions

Human CRC cell HCT116, SW480 and DLD1 were purchased from Shanghai Institute of Cell Biology, Chinese Academy of Sciences (Shanghai, China). HCT116 and SW480 were cultured in Dulbecco’s Modified Eagle’s Medium (Gibco, Logan, UT, USA), DLD1 were maintained in RPMI 1640 Medium (Gibco) in a thermostatic incubator at 37 °C with 5% CO_2_. Medium were added with 10% fetal bovine serum (Gibco) for regular culture.

### Transfection and stable cell lines construction

Cell transfection with small interfering RNA (siRNA) was performed using Lipofectamine 3000 reagent (Invitrogen, Carlsbad, CA, USA). Two independent siRNAs for hnRNPA2B1 and negative control (RiboBio, Guangzhou, China) were introduced into cells according to the manufacturer’s instructions. For construction of stable H19 overexpression and control cell lines, full length H19 (NR-2196.2) and empty lentiviral vector control were synthesized and cloned into a lentiviral vector pEZ-Lv201 (Genecopeia, Guangzhou, China) named pEZ-Lv201-H19 and pEZ-Lv201-Vector respectively, and transfected into HCT116 and SW480 cells. After 72 h, 1μg/ml puromycin was add for 4 weeks to establish stable cell lines. To construct stable H19 knockdown cell lines, two pairs of cDNA oligonucleotides suppressing H19 were cloned into the lentiviral vector psi-LVRU6GP (Genecopeia), called sh-H19–1 and sh-H19–2. A scrambled shRNA was employed as negative control and named sh-NC. After transfected sh-H19–1, sh-H19–2 and sh-NC into HCT116 and DLD1 cells lines, cells were selected with 1μg/ml puromycin for 4 weeks to construct stable cell lines. For construction of overexpressed Raf-1 cell lines, full length Raf-1(NM-2880.3) was cloned into pEZ-M02 vector and a vector with eGFP was used as control (Genecopeia). Plasmid was transfected by using Lipofectamine 3000 reagent (Invitrogen). The siRNA and shRNA sequences are listed in Supplementary Table S1 and S2.

### RNA isolation and quantitative real-time PCR (qRT-PCR)

Total RNA was isolated using RNAiso Plus (Takara, Dalian, China) according to the manufacturer’s instruction. Separation of nuclear and cytoplasm RNA were by using PARIS™ Kit (Life Technologies, Gaithersburg, MD, USA) following the manufacturer’s instruction. Next, reverse transcription was carried out using the PrimeScript™ RT Master Mix (Takara) following the manufacturer’s instruction. Quantitative real-time PCR (qRT-PCR) assays were performed using TB Green® Premix Ex Taq™ II (Takara) by ABI 7900HT Fast RealTime PCR System (Applied Biosystems, Foster City, CA, USA) according to the manufacturers’ protocols. GAPDH was used as an endogenous reference for indicated genes to normalize. To detect RNA in different cellular fractionation, the expression of β-actin was used as cytoplasmic control and U6 as nuclear control. Sequences of primers used for qRT-PCR in this study were shown in Supplementary Table S3.

### Western blot analysis

Total proteins were isolated by using RIPA supplemented with protease and phosphatase inhibitor reagents (Thermo-Fisher Scientific, Waltham, MA, USA). Nuclear and cytoplasm proteins were isolated by PARIS™ Kit (Life) according to the manufacturer’s instruction. Protein was separated using sodium dodecyl sulfate-polyacrylamide gel electrophoresis (SDS-PAGE). After transferred proteins to PVDF membranes (Merck Millipore, Billerica, MA, USA), membranes were blocked in 5% skim milk for 1 h at room temperature followed by incubated with primary antibodies overnight at 4 °C. After three washes with TBST, PVDF membranes were incubated with HRP-conjugated goat anti-mouse (#7076, Cell signaling Technology, Danvers, MA, USA) or anti-rabbit (#7074, CST) secondary antibodies for 1 h at room temperature. Anti-GAPDH antibody (#5174), anti-Tubulin antibody (#2146), anti-Lamin B1 antibody (#13435), anti-Snail antibody (#3879), anti-E-cadherin antibody (#3195), anti-N-cadherin antibody (#13116), anti-ERK1/2 antibody (#9102), anti-Phospho-ERK1/2 antibody (#9101), anti-Raf-1 antibody (#9422) were from Cell Signaling Technology; anti-hnRNPA2B1 antibody (ab6102) was from Abcam (Cambridge, MA, USA).

### Cell migration and invasion assays

The capability of migration and invasion in indicated cancer cells were evaluated by transwell assays. Briefly, for invasion assay, 5 × 10^4 cells were suspended in serum-free medium and seed into the upper chamber (8-μm pore size, BD Biosciences, San Jose, CA, USA)) with diluted Matrigel (Corning, NY, USA). Medium supplemented plus 20% fetal bovine serum was added to the lower chambers. After incubation for 48 h, wiped off cells remained in the upper chamber. Then fixed cells with 4% paraformaldehyde followed by stained with 0.1% crystal violet. The cells migrated or invaded to lower chamber were counted and imaged in three different fields with a microscope.

### Pharmaceuticals

The ERK1/2 inhibitor SCH772984 was purchased from Selleck Chemicals (S7101, Houston, TX, USA). Exponential growing cells seeded in 6-well plates were treated with 0.5 uM SCH772984 for 24 h, and total protein was isolated as described above.

### RNA pull-down assay

Full-length H19 and its antisense RNA were transcribed in vitro using TranscriptAid T7 High Yield Transcription Kit (#K0441, Thermo), treated with RNase-free DNase I (Takara), and purified with GeneJET RNA Purification Kit (#K0731, Thermo) according to the manufacturer’s instruction. Then, RNAs were labeled with desthiobiotinylate using PierceTM RNA 3′end Desthiobiotinylation Kit (#20163, Thermo). RNA pull-down assay was performed using PierceTM Magnetic RNA-protein Pull Down Kit (#20164, Thermo) according to the manufacturer’s instruction. Finally, the RNA-binding proteins were analyzed by liquid chromatography-mass spectrometry (LC-MS) (Triple TOF 6600 LC-MS, AB SCIEX, USA) or followed by western blot.

### RNA immunoprecipitation (RIP)

EZ-Magna RIP RNA Binding Protein Immunoprecipitation Kit (17–700, Millipore) was used to perform RIP assays following the protocol. Anti-hnRNPA2B1 (ab6102, Abcam) antibody and normal mouse Ig (GCS200621, Millipore) were used to immunoprecipitated with target RNA or as a negative control. Finally, RNA was subjected to qRT-PCR analysis as described above to detect the enrichment between indicated RNA binding to hnRNPA2B1.

### Immunofluorescence (IF) assay

Cancer cells were seeded onto sterile slides into 24-well culture plates. Then, fixed cells with 4% paraformaldehyde for 20 min when reached a confluence of 80%. Following permeabilized membranes with 0.1% Triton X-100 for 10 min, blocked antigens with 10% goat serum for 30 min in room temperature. Cells were then incubated with anti-hnRNPA2B1 (ab6102, Abcam) at a 1:100 dilution overnight at 4 °C, followed by further incubation at room temperature for 1 h with AlexaFluor Plus 555 goat anti-mouse IgG secondary antibody (A32727, Thermo) and then labeled DNA with DAPI for 10 min.

### RNA stability

Exponential growing cells were treated with 2.5μg/ml actinomycin D (Act-D, #A9415, Sigma-Aldrich, St. Louis, MO, USA) and incubated at the indicated times. Then RNA was isolated as described above for qRT-PCR. GAPDH was used for normalization.

### Wound healing assay

Cancer cells were seeded into six-well culture plates and cultured until reach 80–90% confluence. Then scratched cells off in a straight line by sterile 100-μl pipette tips and replaced media with serum-free media. Images were recorded by using microscope 48 h after the initial scratches and calculated the distance of wound healing compared to 0 h.

### Cell counting Kit-8 (CCK-8) assay

Cell proliferation was detected by CCK-8 reagent (CK04, Dojindo, Japan). 1000 indicated cells were inoculated into 96-well plates with complete medium for 0 (when cells were adhered), 24. 48 and 72 h. Replaced medium with medium added 10% CCK-8 and incubated for 2 h at 37 °C. Proliferation of cells were measured by using a microplate reader (Multiskan FC, Thermo) at a 450 nm wavelength.

### Animal study

To establish lung metastasis model, female BALB/c nude mice were purchased from Laboratory Animal Center of Sun Yat-sen University (Guangzhou, China). Mice were bred and maintained in a pathogen-free facility until the age of 6-week. 1 × 10^6 H19 overexpressed or control cells were suspended in 100 μL PBS and injected into the tail vein. Experiments were terminated and mice were sacrificed 6 weeks after injection. The lungs from the two groups were anatomized, fixed in formalin, paraffin embedded then analyzed with hematoxylin and eosin (HE) staining to confirm metastatic foci. The handling of mice was strictly under the approval of committee on the Ethics of Animal Experiments of The First Affiliated Hospital, Sun Yat-sen University.

### Statistical analysis

Statistical analyses were performed using SPSS Statistics software version 18.0 software (IBM SPSS Statistics Company, Armonk, NY, USA). All data are presented as the mean ± SD. Appropriate statistical methods including Student’s t-test, Wilcoxon signed-rank test, Mann-Whitney test, Pearson chi-square test were used to calculate differences between groups. Spearman’s correlation analysis was assessed correlation between genes. The Kaplan–Meier method with the log-rank test was used to calculate survival rate between groups. *P*-values < 0.05 was considered to be statistically significant.

## Results

### H19 is upregulated in colorectal cancer and associated with poor survival outcomes

**Fig. 1:**
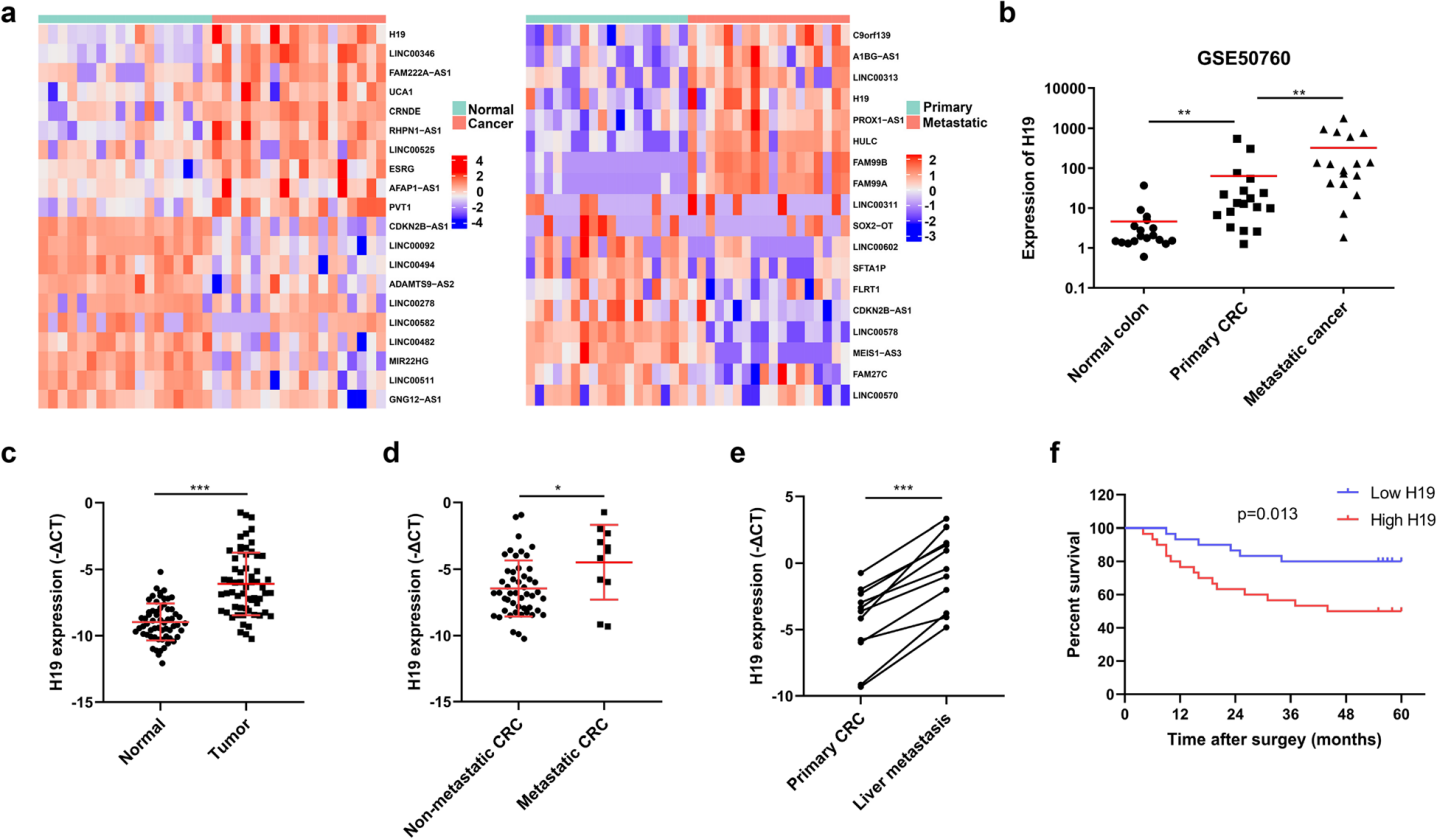
The expression of H19 is upregulated in metastatic CRC and associated with poor prognosis. **a** Left. Heat-maps of top 10 lncRNAs upregulated or downregulated between tumor and matched adjacent normal samples form GSE50760. Right. Heat-maps of lncRNAs changed most substantially between primary tumors and matched metastatic tissues. **b** The expression levels of H19 in matched normal colonic epithelium, primary CRC and metastatic liver lesions from GSE50760. Wilcoxon signed-rank test. **c** Analysis of H19 expression by qRT-PCR in tumors and matched adjacent colonic epithelium tissues of 60 CRC patients from the first affiliated hospital of Sun Yat-sen university. The gene expression is normalized to GAPDH. Student’s t-test. **d** Analysis of H19 expression in primary tumors between patients with (*n* = 11) and without metastasis(*n* = 49) by qRT-PCR. Student’s t-test. **e** Analysis of H19 expression between paired primary tumors and liver metastasis tissues (*n* = 11) by qRT-PCR. Student’s t-test. **f** High H19 expression in CRC correlated with decreased overall survival by Kaplan-Meier survival analysis. The median expression of H19 was used as cut-off. Log-rank test. *P < 0.05, **P < 0.01, ***P < 0.001

To screen the potential lncRNA crucial for metastasis in colorectal cancer, we analyzed the public RNA-seq dataset including 18 colorectal cancer patients with matched normal colonic epithelium, primary lesion and liver metastases tissues (GSE50760) [22]. H19 was one of the most substantially changed lncRNA between normal colonic epithelium and paired CRC tissues. Further analysis of expression of lncRNA between primary CRCs and liver metastasis tissues demonstrated that H19 is also overexpressed compared with that in primary tumors (Fig. 1a and b). To validate the expression pattern of H19, we analyzed 60 primary CRC specimens and corresponding adjacent non-tumor tissues from the tissue bank of the first affiliated hospital of Sun Yat-sen university by qRT-PCR. Our data suggested that H19 is also upregulated in primary tumors compared with paired colonic epithelium tissues (Fig. 1c). To confirmed the association between H19 and clinicopathologic features of CRC, these clinical samples were divided into two group according to the median expression of H19 in primary lesions. High H19 expression was associated with more lymph node metastasis and distant metastasis (Table 1). The expression of H19 in samples with (*n* = 11) or without (*n* = 49) metastasis was measured to elucidate the relationship between H19 and distant metastasis. And H19 was remarkably increased in primary lesions with metastasis compared to those without metastasis (Fig. 1d). Furthermore, we collected and evaluated the expression of H19 in the matched liver metastatic tissues. The expression of H19 in liver metastases was higher than that in matched primary tumors (Fig. 1e). The Kaplan-Meier analysis and log-rank test suggested that CRC patients with high H19 expression was corelated with decreased overall survival (Fig. 1f). Taken together, these data concluded that H19 overexpression was correlated with CRC distance metastasis and poor prognosis.

**Table 1:** Correlation between H19 expression and clinicopathologic features of CRC

Features	N of cases	H19		*p*-value
		Low	High	
Total	60	30	30	
Age (years)				0.795
> 65	34	18	16	
≤ 65	26	12	14	
Gender				0.438
Male	32	14	18	
Female	28	16	12	
Tumor size (cm)				0.119
> 3	33	13	20	
≤ 3	27	17	10	
Depth of invasion				0.613
T1	3	1	2	
T2	7	4	3	
T3	30	17	13	
T4	20	8	12	
Lymph node metastasis				**0.010**
N0	33	19	14	
N1	16	10	6	
N2	11	1	10	
Distant metastasis				**0.042**
M0	49	28	21	
M1	11	2	9	
AJCC stage				0.127
I	8	4	4	
II	25	15	10	
III	16	9	7	
IV	11	2	9	

The median expression level of H19 was used as cut-off

*P*-value was acquired by Pearson chi-square tests

### H19 promotes CRC migration, invasion and EMT in vitro and in vivo

**Fig. 2:**
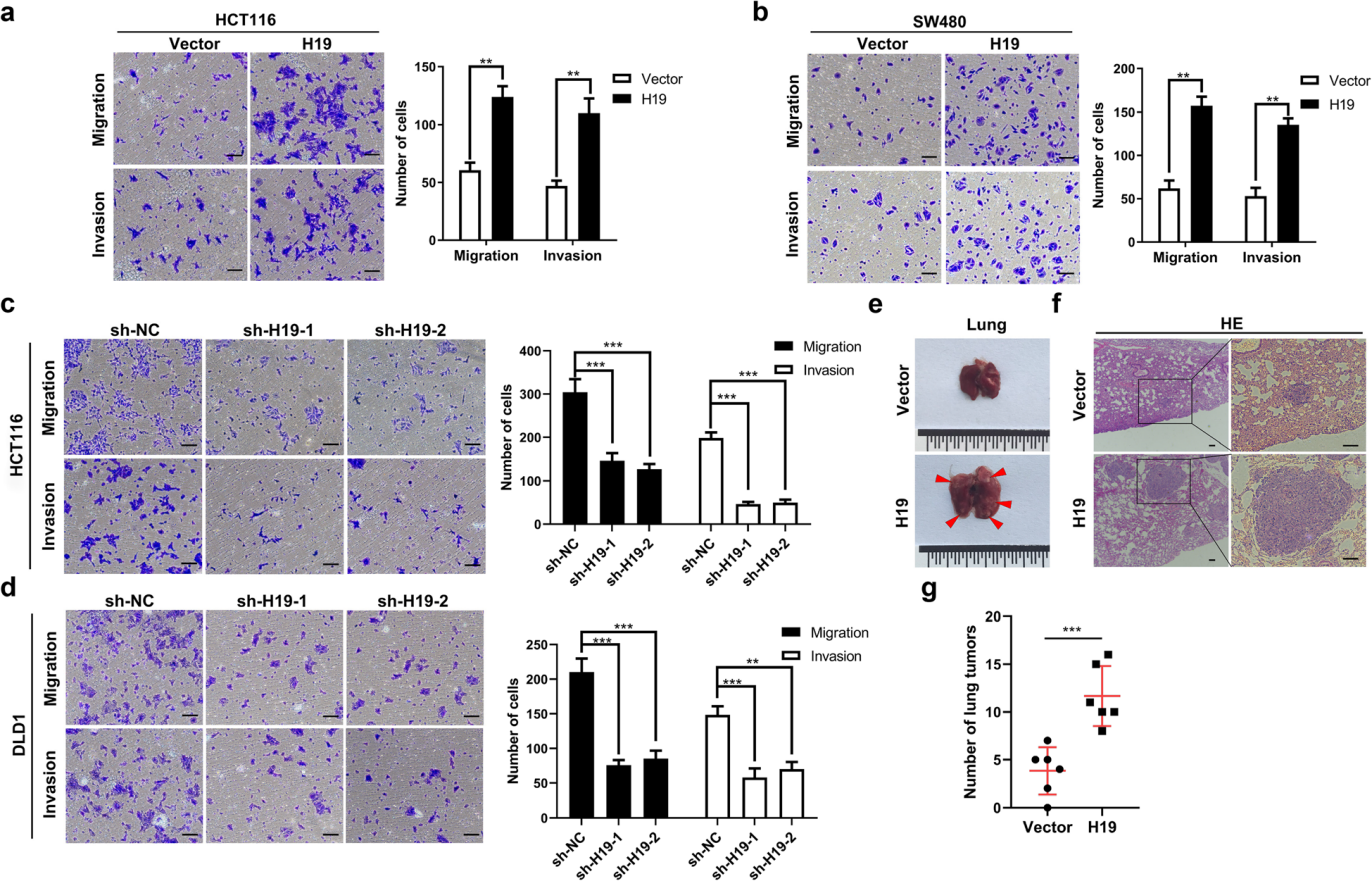
H19 promotes the metastasis of colorectal cancer cells in vitro and in vivo. **a** & **b** Overexpressed H19 promoted the migration and invasion of HCT116 and SW480 cells. **c** & **d** Knockdown of H19 inhibited the migration and invasion of HCT116 and DLD1 cells. **e** The Representative images of metastatic lung tumors after injection of HCT116-H19 and HCT116-Vector cells via tail vein in nude mice. Arrows represent metastatic tumors. **f** HE staining of metastatic lung tumors. **g** The number of metastatic tumors in the lungs of nude mice after injection of HCT116-H19 and HCT116-Vector cells. Scales bars = 100um. Student’s t-test. *P < 0.05, **P < 0.01, ***P < 0.001

To investigate the function of H19 in CRC metastasis, we transfected LV-H19 and control LV-vector to construct H19 stably overexpression HCT116 and SW480 cells (Fig. S1 and S2a). Meanwhile, two independent shRNAs against H19 were transfected into HCT116 and DLD1 cells to establish H19 stably knockdown cells (Fig. S2b). Transwell assays suggested that H19 overexpression promote the migration and invasion of HCT116 and SW480 cells (Fig. 2a and b), whereas knockdown of H19 inhibited migration and invasion of HCT116 and DLD1 cells (Fig. 2c and d). The wound healing assay also confirmed that H19 overexpression promoted cell migration and H19 depletion inhibited cell motility (Fig. S3a and S3b). To further investigate the role of H19 in metastases in vivo, stably overexpressed and control HCT116 cells were injected into BALB/c nude mice through the tail vein to establish lung metastasis model. Our results revealed that overexpression of H19 in HCT116 cells lead to more metastatic tumors in lung and larger tumor size compared to control group (Fig. 2e-g). In addition, CCK-8 analysis demonstrated upregulated or depleted H19 had no significant effect on cell proliferation of CRC cell lines (Fig. S4a and S4b). These results suggested that H19 can contribute to the dissemination of CRC cells in vitro and in vivo.

**Fig. 3:**
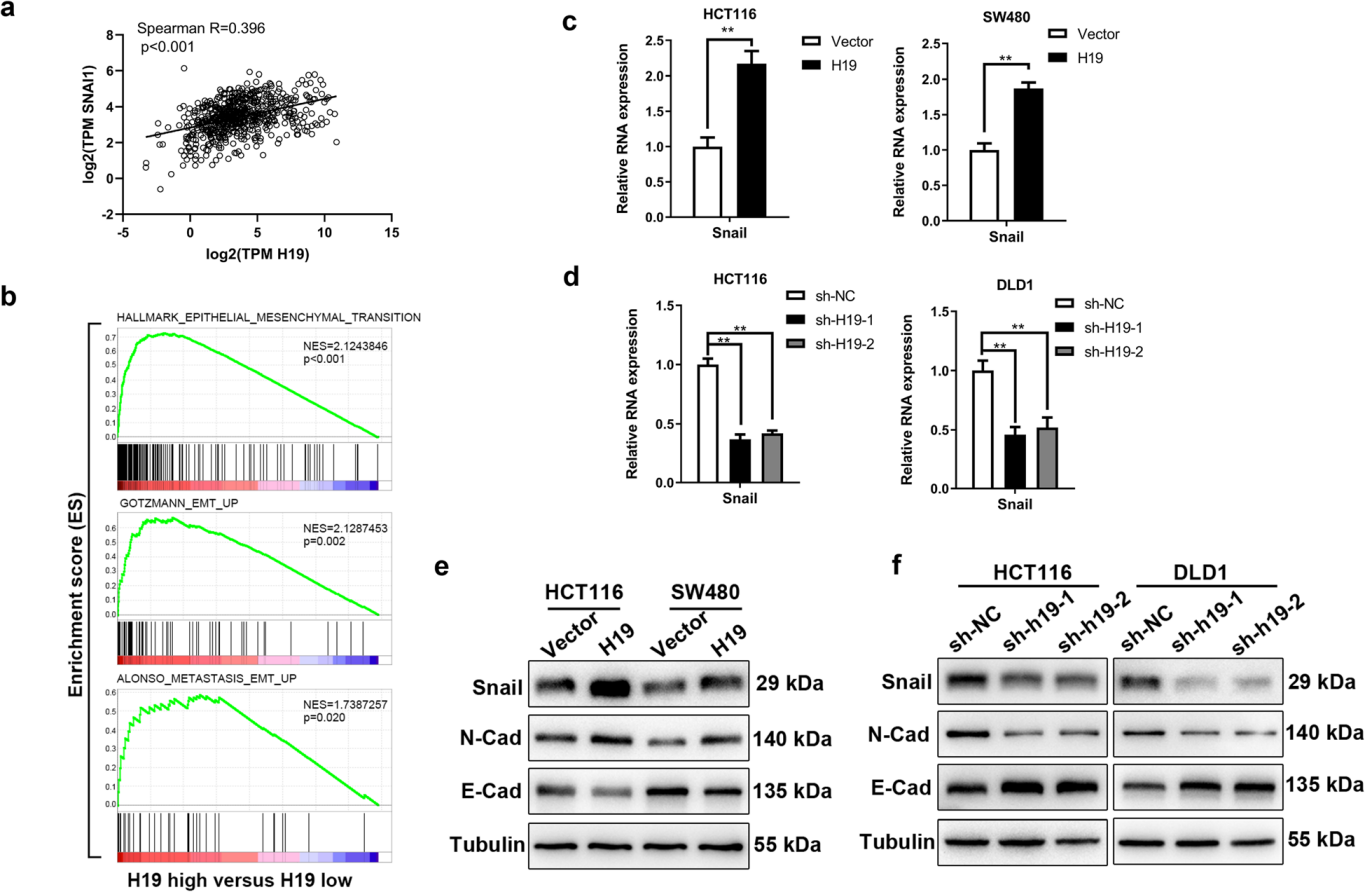
Overexpression of H19 promotes EMT in colorectal cancer cells. **a** Correlation analysis of H19 and Snail expression in TCGA database. **b** GSEA of EMT gene signatures in colorectal cancer samples with high H19 expression versus those with low H19 expression in TCGA database. The median expression of H19 was used as cut-off. **c** Effect of forced expression of H19 on the mRNA level of Snail in HCT116 and SW480 cells were measured by qRT-PCR. **d** Effect of H19 knockdown on the mRNA level of Snail in HCT116 or DLD1 H19 cells were quantified by qRT-PCR. **e** Western blot analysis of EMT markers by ectopic expression of H19 in HCT116 and SW480 cells. **f** Western blot analysis of EMT markers in H19 knockdown or control cells. Student’s t-test. *P < 0.05, **P < 0.01, ***P < 0.001

To further investigate how H19 regulate metastasis in colorectal cancer, correlation analysis was performed to confirm the related genes of H19 in The Cancer Genome Atlas (TCGA) colorectal adenocarcinoma database. The result displayed that SNAI1 (Snail), a crucial EMT transcript factor, was one of the most relevant genes with H19 (Fig. 3a and S5), suggested H19 may involve in the progression of EMT in colorectal cancer. In addition, Gene Set Enrichment Analysis (GSEA) in gene expression profiles of colorectal cancer patients obtained from the TCGA database indicated that EMT pathway from the Molecular Signatures Database [23] and two published EMT gene signatures [24, 25] were significantly enriched in samples with high level of H19, demonstrated H19 is highly correlated with EMT signaling (Fig. 3b). To validated the association between H19 and EMT in colorectal cancer, we first confirmed the influence of H19 on the expression of EMT transcript factors in CRC cells because of their direct functions to induce EMT. RT-PCR data suggested that H19 overexpression increase the mRNA level of Snail but not Slug, Smuc, Zeb1, Zeb2, Twist1, Twist2 or E12/E47 in HCT116 and SW480 cells (Fig. 3c and S6a). Consistently, H19 depletion reduce the expression of Snail but not Slug, Smuc, Zeb1, Zeb2, Twist1, Twist2 or E12/E47 in HCT116 and DLD1 cells (Fig. 3d and S6b). Western blot result indicated H19 overexpression increased the expression of Snail, N-cadherin and inhibited E-cadherin expression (Fig. 3e), whereas H19 depletion downregulated Snail, N-cadherin expression and upregulated the expression of E-cadherin (Fig. 3f). Hence, these results suggested that H19 promote EMT, migration, invasion and metastasis of CRC cells.

### H19 specifically binds to hnRNPA2B1

**Fig. 4:**
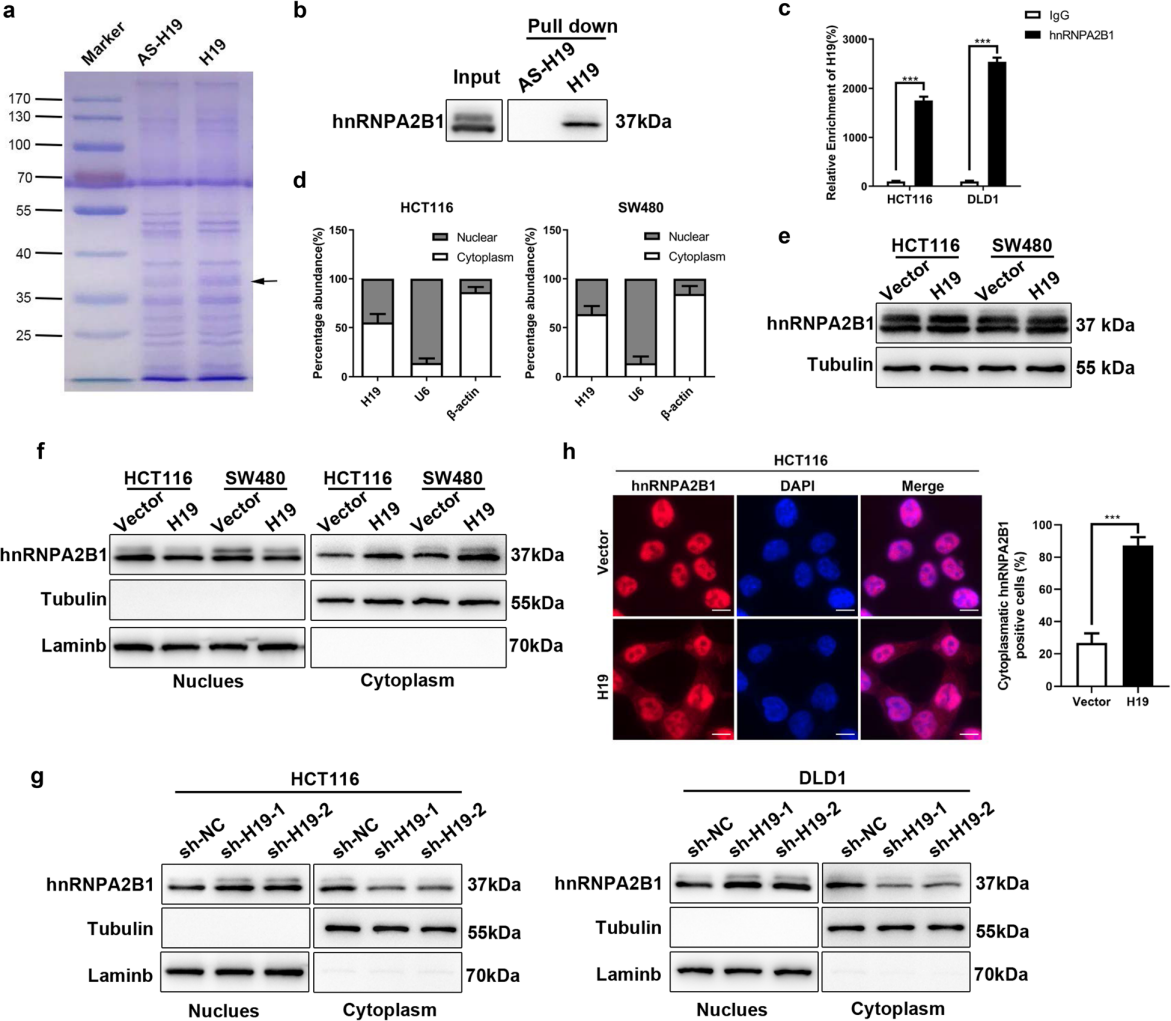
H19 binds to hnRNPA2B1 specially. **a** Coomassie blue staining of gel after SDS-PAGE separated the proteins which were immunoprecipitated with full length of H19 and its antisense RNA in HCT116 cells by RNA pull-down assays. The arrow shows the position of hnRNPA2B1. **b** Western blot analyses following RNA pull-down assays in HCT116 cells confirmed the interaction between H19 and hnRNPA2B1. Input, total proteins. Pull down, proteins immunoprecipitated by RNA. **c** RIP assay followed by qRT-PCR suggested H19 binds to hnRNPAA2B1. **d** qRT-PCR to analyze the subcellular distribution of H19 after compartmentalization of cytoplasmic and nuclear fractions in HCT116 and DLD1 cells. β-actin and U6 act as cytoplasmic and nuclear controls, respectively. **e** Western blot to analyze total level of hnRNPA2B1 between H19 overexpressed and control cells. **f** Western blot analyzed the effect of H19 on the protein level of hnRNPA2B1 in nuclear and cytoplasm. **g** Western blot analyzed the protein level of hnRNPA2B1 in nuclear and cytoplasm of H19 depleted and control cells. **h** Immunofluorescence were performed to investigate the subcellular localization of hnRNPA2B1 of H19 overexpression and control cells. Scales bars = 10um Student’s t-test. *P < 0.05, **P < 0.01, ***P < 0.001

Since lncRNA can implement their functions through binding to proteins [26, 27], RNA pull-down assays were performed followed by LC-MS to detect the target of H19 (Figs. 4a, S7 and Table S4). The result demonstrated that hnRNPA2B1 was one of the most abundant proteins interacting with H19, which has been reported to regulate EMT and metastasis in tumors [28]. Hence, hnRNPA2B1 were selected as candidates for subsequent mechanistic experiments. First, western blot followed RNA pull-down assays confirmed the capability of hnRNPA2B1 binding to H19 in HCT116 cells (Fig. 4b). Consistently, RNA immunoprecipitation assays suggested that the enrichment of H19 precipitated by antibodies against hnRNPA2B1 increased greatly compared with those by control IgG in HCT116 and DLD1 cells, which further validated the interactions between H19 and hnRNPA2B1 (Fig. 4c). To further investigate the role of hnRNPA2B1 in H19-regulated metastasis in colorectal cancer, we separated the nuclear fractionation and cytoplasm fractionation of HCT116 and SW480 cells to investigate the distribution of H19 in subcellular fraction. RT-PCR demonstrated H19 distributes in cytoplasm as well as in nucleus (Fig. 4d), suggested H19 could implement its functions at the transcriptional or post-transcriptional level. Next, we tried to elucidate whether H19 can regulate the expression of hnRNPA2B1 through their interactions or not. Western blot result indicated H19 overexpression did not influence total protein level of hnRNPA2B1 in HCT116 or SW480 cells (Fig. 4e). It has been reported that some hnRNPs shuttle between the nucleus and the cytoplasm [29], and consequently facilitated its binding to the target RNA [30]. Thus, the distribution of hnRNPA2B1 in H19 overexpression and control colorectal cancer cells were compared. Our results demonstrated H19 overexpression enhance the enrichment of hnRNPA2B1 in the cytoplasm (Fig. 4f), while knockdown of H19 decrease cytoplasmic hnRNPA2B1 (Fig. 4g). Furthermore, immunofluorescence also confirmed H19 overexpression increase the protein level of hnRNPA2B1 in cytoplasm (Fig. 4h) and H19 depleted reduce the enrichment of hnRNPA2B1 in cytoplasm (Fig. S8). Taken together, these data suggested that H19 specifically binds to hnRNPA2B1 and promote its translocation from nucleus to cytoplasm.

### H19 regulates EMT through hnRNPA2B1 dependent ERK pathway

**Fig. 5:**
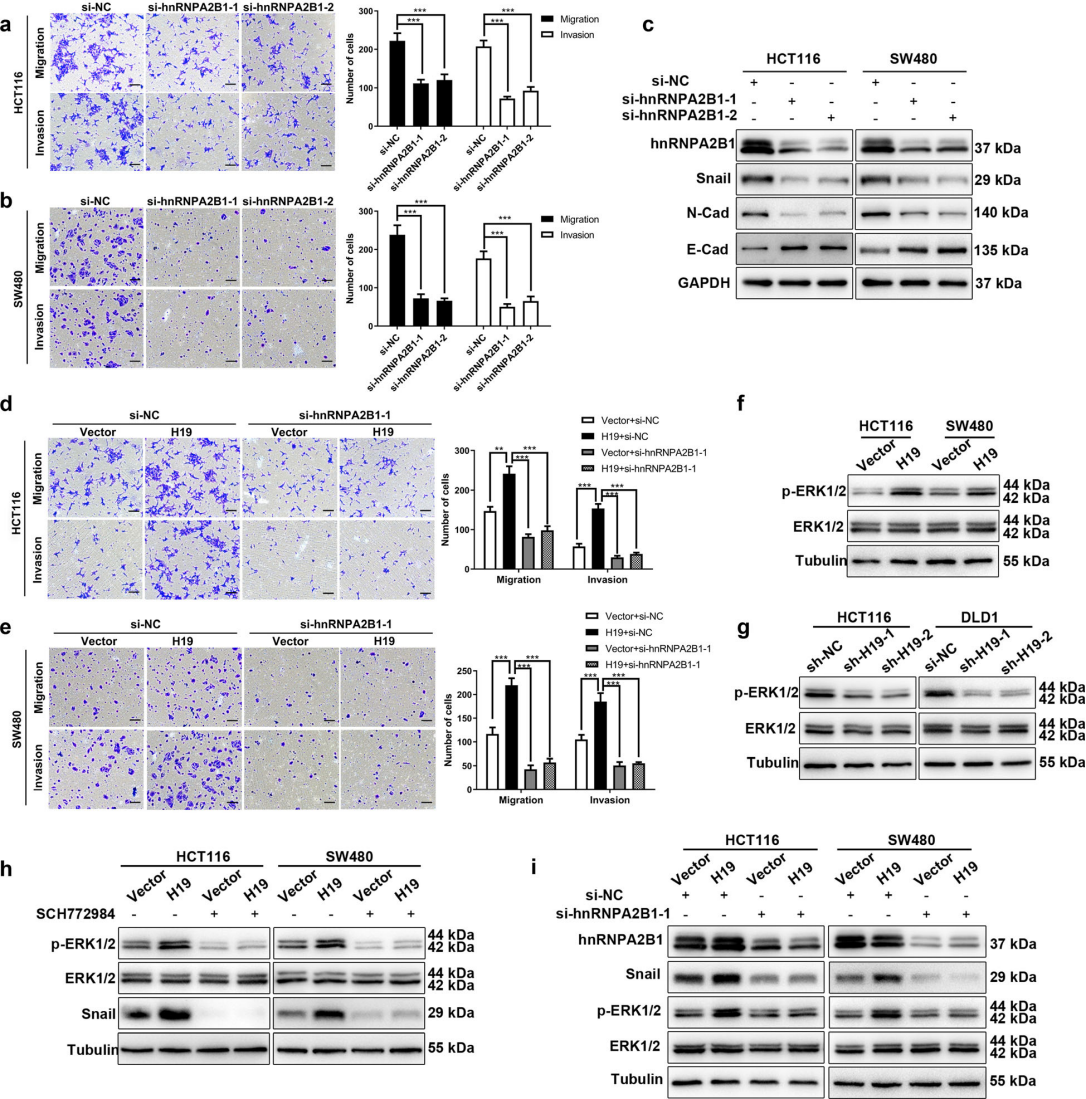
H19 induced migration and invasion in colorectal cancer through hnRNPA2B1 **a** HnRNPA2B1 knockdown blocked the migration and invasion of HCT116 cells. **b** HnRNPA2B1 knockdown decreased the migration and invasion of SW480 cells. **c** Western blot analyses of EMT markers (Snail, E-cadherin and N-cadherin) after silencing of hnRNPA2B1in HCT116 and SW480 cells. **d** Knockdown of hnRNPA2B1 inhibited H19-induced migration and invasion in HCT116 cells. **e** Silencing of hnRNPA2B1 decreased H19-induced migration and invasion in SW480 cells. **f** Ectopic expression of H19 elevated the phosphorylation of ERK in colorectal cancer cells. **g** Western blot showed altered levels of p-ERK after H19 knockdown in HCT116 and DLD1 cells. **h** SCH772984 decreased the phosphorylation level of p-ERK, and the expression of Snail in HCT116-H19 and SW480-H19 cells. **i** HnRNPA2B1 knockdown reversed H19-induced p-ERK and Snail expression. Scales bars = 100um. Student’s t-test. *P < 0.05, **P < 0.01, ***P < 0.001

Next the effect of hnRNPA2B1 in metastases were explored to test whether it is a downstream mechanism of H19. Knockdown of hnRNPA2B1 inhibited the migration and invasion of HCT116 and SW480 cells (Fig. 5a and b) as well as inhibited EMT (Fig. 5c), suggested a positive role, similar as H19, in regulating EMT. Next, we investigate whether H19 promote EMT and metastasis depending on hnRNPA2B1 in CRC. Silencing of hnRNPA2B1 in HCT116-H19 or SW480-H19 cells attenuated H19-induced migration and invasion (Fig. 5d and e). These results suggested that hnRNPA2B1 contribute to the invasiveness of colorectal cancer cells induced by H19. Emerging evidence demonstrated that hnRNPA2B1 promotes tumor metastasis through extracellular regulated protein kinases (ERK) pathway [31]. We therefore investigated the functions of H19 and hnRNPA2B1 in ERK pathway. Western blot analysis demonstrated that H19 overexpression elevate the phosphorylation of ERK (Fig. 5f), while H19 knockdown lead to decreased phosphorylation of ERK (Fig. 5g). In addition, SCH772984, an ERK pathway inhibitor, attenuated the upregulation of Snail in HCT116-H19 and SW480-H19 cells (Fig. 5h). These results indicated that H19 regulate the expression of Snail through the activation of ERK signaling. Furthermore, knockdown of hnRNPA2B1 reversed the phosphorylation of ERK and upregulation of Snail in colorectal cancer cells with stable H19 overexpression (Fig. 5i). Collectively, our results demonstrated that H19 overexpression can lead to activation of ERK signaling through hnRNPA2B1, and eventually induced EMT and metastasis in CRC.

### H19 actives ERK signaling by upregulating Raf-1 expression

**Fig. 6:**
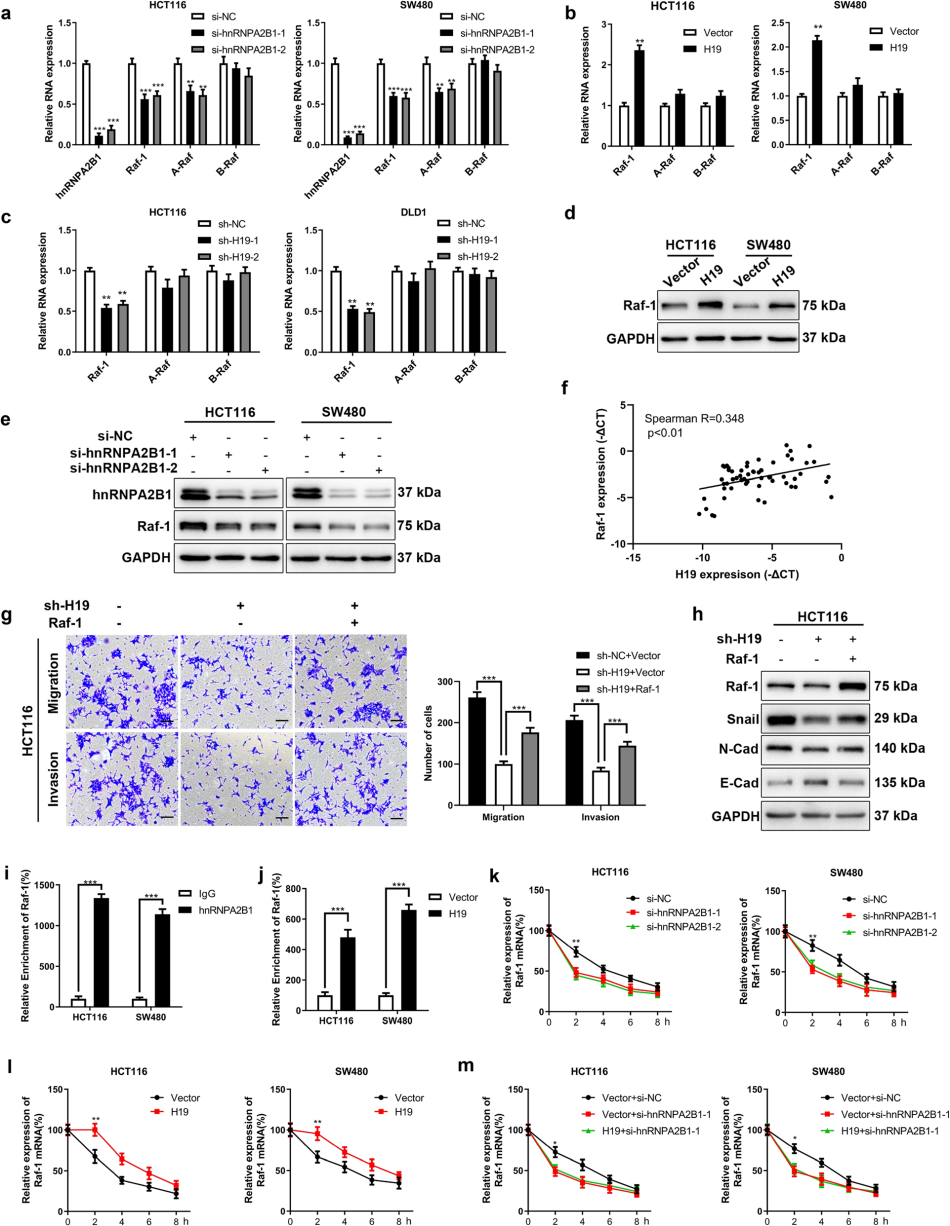
H19 stabilize RAF1 by interacting with hnRNPA2B1. **a** The effect of hnRNPA2B1 knockdown on the mRNA level of Raf kinase family was measured by qRT-PCR. **b** The influence of H19 overexpression on the mRNA level of Raf kinase family were measured by qRT-PCR in colorectal cancer cells. **c** The impact of H19 knockdown on the mRNA level of Raf kinase family were measured by qRT-PCR in colorectal cancer cells. **d** Western blot analysis of Raf-1 in H19 stable overexpression or control cells. **e** Western blot analysis of Raf-1 after hnRNPA2B1 knockdown in HCT116 and SW480 cells. **f** Correlation analysis of H19 and Raf-1 in primary colorectal cancer tissues. **g** Overexpression of Raf-1 rescued the migration and invasion of H19-depleted HCT116 cells. **h** Western blot analyses of EMT markers after overexpression of Raf-1 in H19-depleted HCT116 cells. **i** RIP assay followed by qRT-PCR explored the enrichment of Raf-1 mRNA binding to hnRNPAA2B1. **j** RIP experiment showed that H19 overexpression increased the enrichment between Raf-1 mRNA and hnrnpA2B1. **k** Knockdown of hnRNPA2B1 disrupted the stability of Raf-1 mRNA compared with the control group. **l** Overexpression of H19 increased the stability of Raf-1 mRNA compared with the control group. **m** Knockdown of hnRNPA2B1 reversed H19-induced Raf-1 mRNA stabilization. Student’s t-test. *P < 0.05, **P < 0.01, ***P < 0.001

We next sought to determine the underlying mechanism how H19 activate ERK signaling through hnRNPA2B1. It has been reported that hnRNPA2B1 regulates the expression and splicing of A-Raf [32], a member of Raf kinase family, which are main activators of ERK signaling [33]. Therefor we explored the function of H19 in regulating the expression of Raf kinase family. Knockdown hnRNPA2B1with siRNA reduced the mRNA level of Raf-1 (C-Raf) and A-Raf, but not B-Raf in HCT116 and SW480 cells (Fig. 6a), whereas the overexpression or silencing of H19 only altered the mRNA level of Raf-1(Fig. 6b and c), suggested Raf-1 may play a critical role in H19-mediated activation of ERK signaling. Furthermore, we found that overexpression of H19 upregulated the protein levels of Raf-1 in HCT116 and SW480 cells (Fig. 6d). Simultaneously, hnRNPA2B1 knockdown reduced expression of Raf-1 (Fig. 6e). These results indicated that H19 and hnRNPA2B1 regulated expression of Raf-1 at RNA level. To further confirmed the association between H19, hnrnpA2B1 and Raf-1, we analyzed the expression of H19, hnrnpA2B1 and Raf-1 in the primary colorectal cancer tissues and demonstrated the expression of H19 is positive correlated with Raf-1 (Figs. 6f, S9a and S9b). The recuse experiment revealed that overexpressed Raf-1 in H19-depleted HCT116 cells upregulated the migration, invasion (Fig. 6g) and EMT (Fig. 6h) of HCT116 cells. Because hnRNPA2B1 stabilizes a variety of its target RNA [34], we suppose H19 upregulates the expression of Raf-1 through the interaction between hnRNPA2B1 and Raf-1 mRNA. To further validate this, RIP assays were performed, and indicated a possible binding between hnRNPA2B1 and Raf-1 mRNA in HCT116 and SW480 cells (Fig. 6i). Next, we investigated the influence of H19 on the interaction between hnRNPA2B1 and Raf-1. RIP assays followed by qRT-PCR suggested that the enrichment of Raf-1 precipitated by antibodies against hnRNPA2B1 in H19 stable overexpression cells were increased significantly compared to that in control cells, revealing H19 facilitates the binding between hnRNPA2B1 and Raf-1 (Fig. 6j). Consistently, H19 knockdown decreased the binding between hnRNPA2B1 and Raf-1 (Fig. S10), suggested the binding between hnRNPA2B1 and Raf-1 mRNA is H19 dependent. Furthermore, by treating cancer cells with Act-D to terminate transcription, our results demonstrated that silencing of hnRNPA2B1 significantly decrease the stability of Raf-1 in HCT116 and SW480 cells (Fig. 6k). Moreover, overexpression of H19 stabilized Raf-1 mRNA (Fig. 6l) while knockdown hnRNPA2B1 in HCT116-H19 and SW480-H19 attenuated the effect of H19 on the stability of Raf-1 (Fig. 6m), indicated H19 regulated Raf-1 expression via hnRNPA2B1 at post-transcript level. All together, these results suggested H19 enhanced the stability of Raf-1 mRNA via hnRNPA2B1, subsequently activated Raf-ERK signaling.

## Discussion

Metastasis causes most cancer-related deaths in patients with colorectal cancer [2]. However, the critical molecules regulating tumor metastasis are still largely unknown. Among the complex process of metastasis development, long non-coding RNAs were identified to play important roles in regulating cancers metastasis [21]. In the present study, we demonstrate H19 promotes the migration, invasion and metastasis of colorectal cancer cells in vitro and in vivo. By binding to hnRNPA2B1, H19 leads to EMT via Raf-ERK dependent signaling, and finally promotes the dissemination of CRC.

LncRNA has been reported to exert crucial influence on the development of cancers [35]. Regarding the metastasis of CRC, few lncRNA have been functionally elucidated. To identified lncRNA related to metastasis in CRC, the expression profile of CRC between primary tumors and metastases in public database were analyzed. H19 was supposed to be one of the top overexpressed lncRNA both in primary tumor and metastatic tissues compared with adjacent normal tissues. Some studies have demonstrated that the overexpression of H19 is associated with increased risk for several malignancies [36, 37]. Among that, H19 was reported to be potential prognostic biomarker to predict liver metastases in CRC patients [38], but the exact mechanism remained unclear. To further confirm the role of H19 in the regulation of CRC metastasis, we analyzed the expression pattern of H19 in clinical specimens. Our results revealed that H19 is upregulated in liver metastases and primary tumors compared with adjacent non-cancerous tissues. In addition, high H19 was associated with more positive lymph node and distance metastasis, and worse survival outcomes, suggesting H19 to be a potential predictor for CRC metastasis and prognosis.

Considering the increased expression and a potential pro-metastasis role of H19 in colorectal cancer, the effect of H19 on the invasiveness of CRC cells were evaluated in vitro. Overexpression of H19 markedly enhanced, whereas H19 silencing reduced the migration and invasion of HCT116 and SW480 cells. On the contrary, the CCK-8 assays found that H19 have no significant effect on the viability of HCT116 and SW480 cells because H19 may mainly influence the cell motility but not viability of colorectal cancer, and further study on the influence of H19 on cell cycle should be carried out to ensure the underlying mechanism of H19. Further analysis in vivo demonstrated that overexpression of H19 leads to more metastases and larger tumor size in lung compared with control cells. Taken together, these results suggested that H19 promoted colorectal cancer metastases in vitro and in vivo, but exerted no influence on cell viability.

Nevertheless, the molecular mechanism of H19 to upregulate the metastatic capability of tumor cells in colorectal cancer were not clear. It is well established that EMT, a transition of cancer cells from epithelial phenotype to gain mesenchymal properties, is a prominent process for cancer metastases [39]. It has been reported that H19 affect EMT by functioning as miRNA sponges in breast cancer [18]. To further investigate the underlying mechanisms for H19 promoting migration and invasion of CRC cells, correlation analysis was performed according to the data in TCGA, and revealed that the expression of H19 is positively correlated with Snail, an EMT- transcription factor, which induce EMT by transcriptionally represses E-cadherin [40]. Our data suggested that ectopic expression of H19 increase the RNA and protein level of Snail, as well as N-cadherin, and decreased the level of E-cadherin. Consistently, H19 knockdown downregulated the expression of Snail and N-cadherin, but increased the level of E-cadherin. Collectively, our study demonstrated that H19 can upregulate Snail expression, which subsequently induced EMT, and eventually promoted CRC metastasis in vitro and in vivo.

**Fig. 7:**
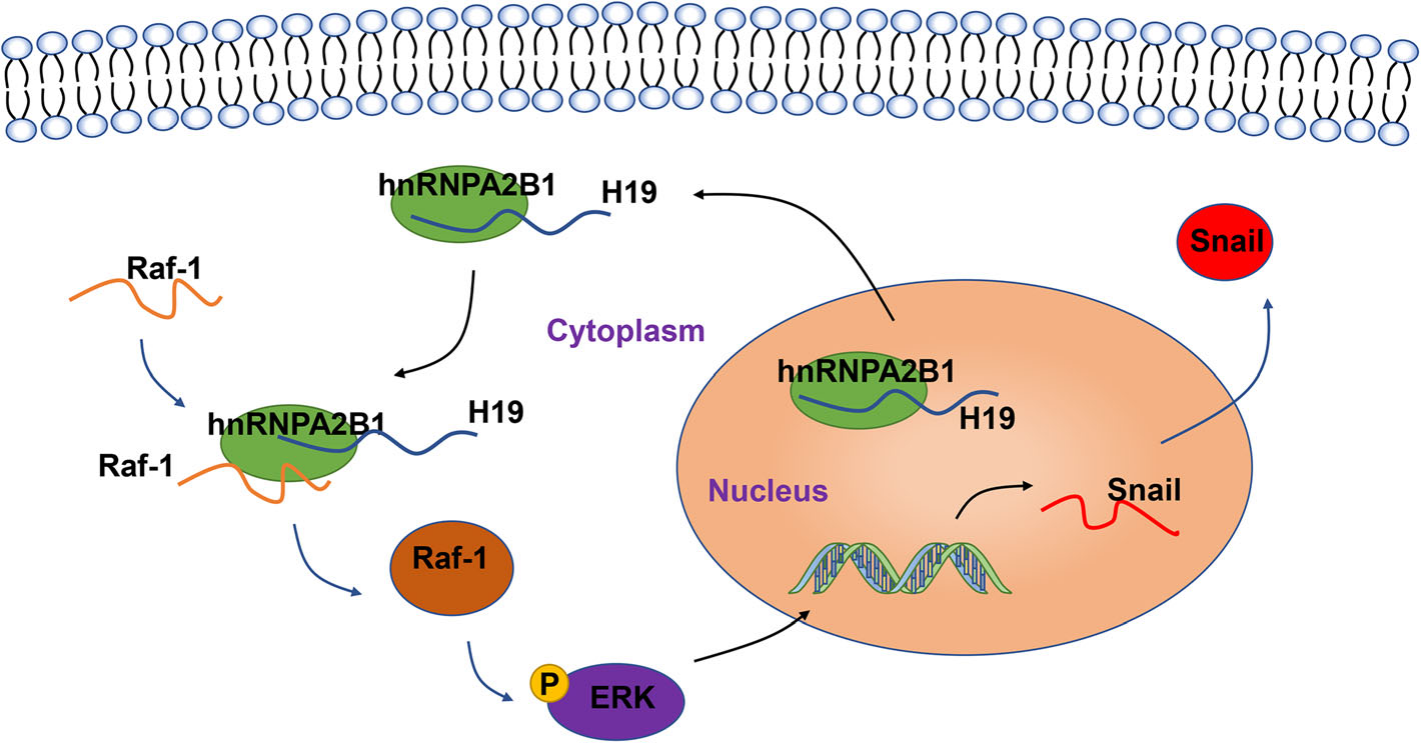
Model of H19 interaction with hnRNPA2B1 and the signaling pathways involved in colorectal cancer metastasis. H19 interacts with hnRNPA2B1, promoting the translocation of hnRNPA2B1 from nucleus to cytoplasm, facilitating the binding between hnRNPA2B1 and Raf-1 mRNA, thereby stabilizing Raf-1 mRNA. Upregulation of Raf-1 activate ERK signaling pathways, thereby promoting the transcription of Snail and inducing EMT

Mounting evidence showed that lncRNA act as key regulators by binding to RNA binding proteins (RBP). To explore the potential RBPs that H19 binds to, RNA pull-down assays and LC-MS were performed, and found that H19 binds to hnRNPA2B1 directly. HnRNPA2B1, a member of heterogeneous nuclear ribonucleoproteins mainly located in the nucleus, are of crucial importance for the stabilization of its target transcripts [34]. It has been reported that lncRNA can regulate the translocation of hnRNPs from nuclear to cytoplasm [30], leading to subsequent biological process in cancer [29]. Several studies have demonstrated cytoplasmic localization of hnRNPA2B1 is associated with oncogenesis [41, 42]. In present study, we found overexpression of H19 triggers the translocation of hnRNPA2B1 from nuclear to cytoplasm. Knockdown of hnRNPA2B1 alleviated the enhanced capability of migration and invasion caused by H19, as well as the expression of Snail, indicated a pivotal role of hnRNPA2B1 in H19-mediated EMT and metastasis. Ras/Raf/MEK/ERK signaling is always aberrantly activated and upregulate the expression of EMT transcript factors, consequently resulting in EMT and metastases in various types of cancers [33, 43, 44]. Our data demonstrated that the overexpression of H19 elevate the phosphorylation of ERK. Meanwhile, ERK signaling inhibitor blocked the upregulation of Snail caused by H19 overexpression, suggested H19 regulates the expression of Snail via ERK pathway. In addition, knockdown of hnRNPA2B1 attenuated the activation of ERK signaling induced by H19, which further confirmed hnRNPA2B1 work as downstream mechanism of H19 to promote EMT. To further elucidate the mechanism of ERK signaling activation, we found overexpression of H19 facilitates the binding between hnRNPA2B1 and the mRNA of Raf-1 and consequently stabilizes Raf-1. Ectopic H19 expression upregulated the expression of Raf-1, subsequently activated Raf-ERK signaling. Overexpressed Raf-1 rescued the migration, invasion and EMT in H19-depleted cells. In addition, hnRNPA2B1 knockdown reversed the effect of H19 on the stability of Raf-1, suggested H19 exert its functions dependent on hnRNPA2B1. Collectively, our study demonstrated the overexpression of H19 triggers the translocation of hnRNPA2B1 from nuclear to cytoplasm and increases the enrichment between hnRNPA2B1 and Raf-1 mRNA, consequently stabilizes and upregulates the expression of Raf-1, eventually leads to activation of Raf-ERK signaling and EMT (Fig. 7).

## Conclusions

In conclusion, our finding reveals that lncRNA H19 is upregulated in colorectal cancer, and correlated with poor outcomes in CRC patients. By directly binding to hnRNPA2B1, H19 activates Raf-ERK signaling, resulting in the induction of EMT, and eventually promotes migration, invasion and metastasis of colorectal cancer cells. Our study discovers the critical role of H19 in mediating the pro-metastatic potential of CRC and highlight the potential of H19 acting as a prognostic predictor and therapeutic target for colorectal cancer.

## Supplementary information

**Additional file 1.**

**Additional file 2.**

**Additional file 3.**

**Additional file 4.**

**Additional file 5.**

**Additional file 6.**

**Additional file 7.**

**Additional file 8.**

**Additional file 9.**

**Additional file 10.**

**Additional file 11: Table S1.** Target sequences of siRNA **Table S2.** Target sequences of shRNA **Table S3.** Sequences of primers

**Additional file 12: Table S4.** Identifed the proteins binding to H19 by LC-MS

**Additional file 13: Figure S1.** The expression of H19 in different colorectal cancer cell lines detected by qRT-PCR. **Figure S2.** a The expression of H19 in HCT116 and SW480 cells after transfected with lv-H19 and control lv-Vector to construct stable H19 overexpression cell lines. b The expression of H19 in HCT116 and DLD1 cells after transfected with sh-NC, sh-H19-1 and sh-H19-2 to construct stable H19 knockdown cell lines. Student’s t-test. *P < 0.05, **P < 0.01, ***P < 0.001 **Figure S3.** a Wound healing assay in HCT116 H19 stable overexpression and control. The relative migration rate is calculated compared to the distance of 0h. b Wound healing assay in H19 knockdown and control DLD1 cells. Scales bars = 250um. Student’s t-test. *P < 0.05, **P < 0.01, ***P < 0.001 **Figure S4.** a CCK-8 assays of H19 overexpression or control cells. b CCK-8 assays of H19 knockdown or control cells. Student’s t-test. *P < 0.05, **P < 0.01, ***P < 0.001 **Figure S5.** Heatmap of the top 50 genes which H19 is most corelated with in TCGA database. **Figure S6.** a Effect of H19 overexpression on the mRNA level of EMT transcript factors in HCT116 and SW480 cells were measured by qRT-PCR. b Effect of H19 knockdown on the mRNA level of EMT transcript factors in HCT116 or DLD1 H19 cells were quantified by qRT-PCR. **Figure S7.** Agarose gel electrophoresis of RNA probe used for RNA pull-down assay. **Figure S8.** Immunofluorescence were performed to investigate the subcellular localization of hnRNPA2B1 in H19 depleted and control cells. Scales bars=10um. **Figure S9.** a Correlation analysis between H19 and hnRNPA2B1. b Correlation analysis between hnRNPA2B1 and Raf-1. **Figure S10.** RIP assay followed by qRT-PCR explored the enrichment of Raf-1 mRNA binding to hnRNPAA2B1 in H19 depleted and control cells. Student’s t-test. *P < 0.05, **P < 0.01, ***P < 0.001

## Data Availability

The datasets from the current study are available from the corresponding author on reasonable request. Publicly available data was obtained from the GEO database (https://www.ncbi.nlm.nih.gov/gds) and TCGA database (https://portal.gdc.cancer.gov/).

## Abbreviations

lncRNA: Long non-coding RNA
CRC: Colorectal cancer
EMT: Epithelial-to-mesenchymal transition
LC-MS: Liquid chromatography-mass spectrometry
hnRNPA2B1: Heterogeneous nuclear ribonucleoprotein A2/B1
qRT-PCR: Quantitative real-time PCR
ERK: Extracellular signal-regulated kinase
RIP: RNA immunoprecipitation
IF: Immunofluorescence
Act-D: Actinomycin D
CCK-8: Cell Counting Kit-8
TCGA: The Cancer Genome Atlas
GSEA: Gene Set Enrichment Analysis
RBP: RNA binding protein
