# Correction to: Long non-coding RNA H19 promotes colorectal cancer metastasis via binding to hnRNPA2B1

**DOI:** 10.1186/s13046-021-01911-z

**Published:** 2021-03-23

**Authors:** Yuhui Zhang, Weibin Huang, Yujie Yuan, Jin Li, Jing Wu, Jie Yu, Yulong He, Zhewei Wei, Changhua Zhang

**Affiliations:** 1 Department of Gastrointestinal Surgery, the First Affiliated Hospital of Sun Yat-sen University, 58 Zhongshan 2nd Road, Guangzhou, 510080 Guangdong China; 2 Center for Digestive Disease, the Seventh Affiliated Hospital of Sun Yat-sen University, 628 Zhenyuan Road, Shenzhen, 518000 Guangdong China

**Correction to: J Exp Clin Cancer Res 39, 141 (2020)**

**https://doi.org/10.1186/s13046-020-01619-6**

Following publication of the original article [1], the authors identified minor errors in image-typesetting in Fig. 2; specifically the migration transwell assay of sh-H19–2 DLD1 cells group displayed in Fig. 2d.

**Fig. 2:**
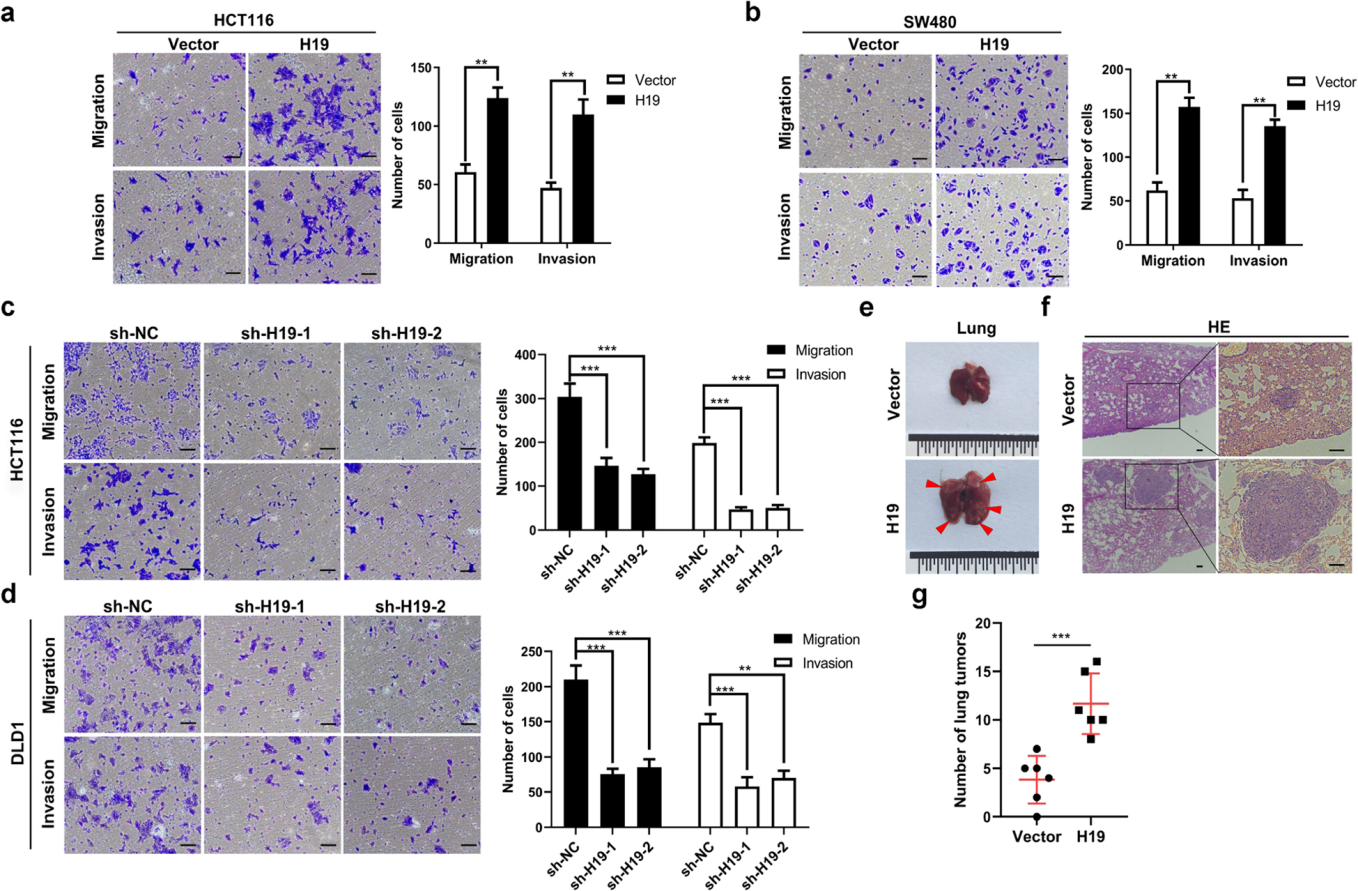
H19 promotes the metastasis of colorectal cancer cells in vitro and in vivo. **a** & **b** Overexpressed H19 promoted the migration and invasion of HCT116 and SW480 cells. **c** & **d** Knockdown of H19 inhibited the migration and invasion of HCT116 and DLD1 cells. **e** The Representative images of metastatic lung tumors after injection of HCT116-H19 and HCT116-Vector cells via tail vein in nude mice. Arrows represent metastatic tumors. **f** HE staining of metastatic lung tumors. **g** The number of metastatic tumors in the lungs of nude mice after injection of HCT116-H19 and HCT116-Vector cells. Scales bars = 100um. Student’s t-test. *P < 0.05, **P < 0.01, ***P < 0.001

The corrected figure is given below. The correction does not have any effect on the results or conclusions of the paper. The original article has been corrected.
