# Supplementary figures and images for: Long non-coding RNA H19 promotes colorectal cancer metastasis via binding to hnRNPA2B1
Source: J Exp Clin Cancer Res. 2020 Jul 23;39:141. doi: 10.1186/s13046-020-01619-6 (PMC7412843; doi:10.1186/s13046-020-01619-6)

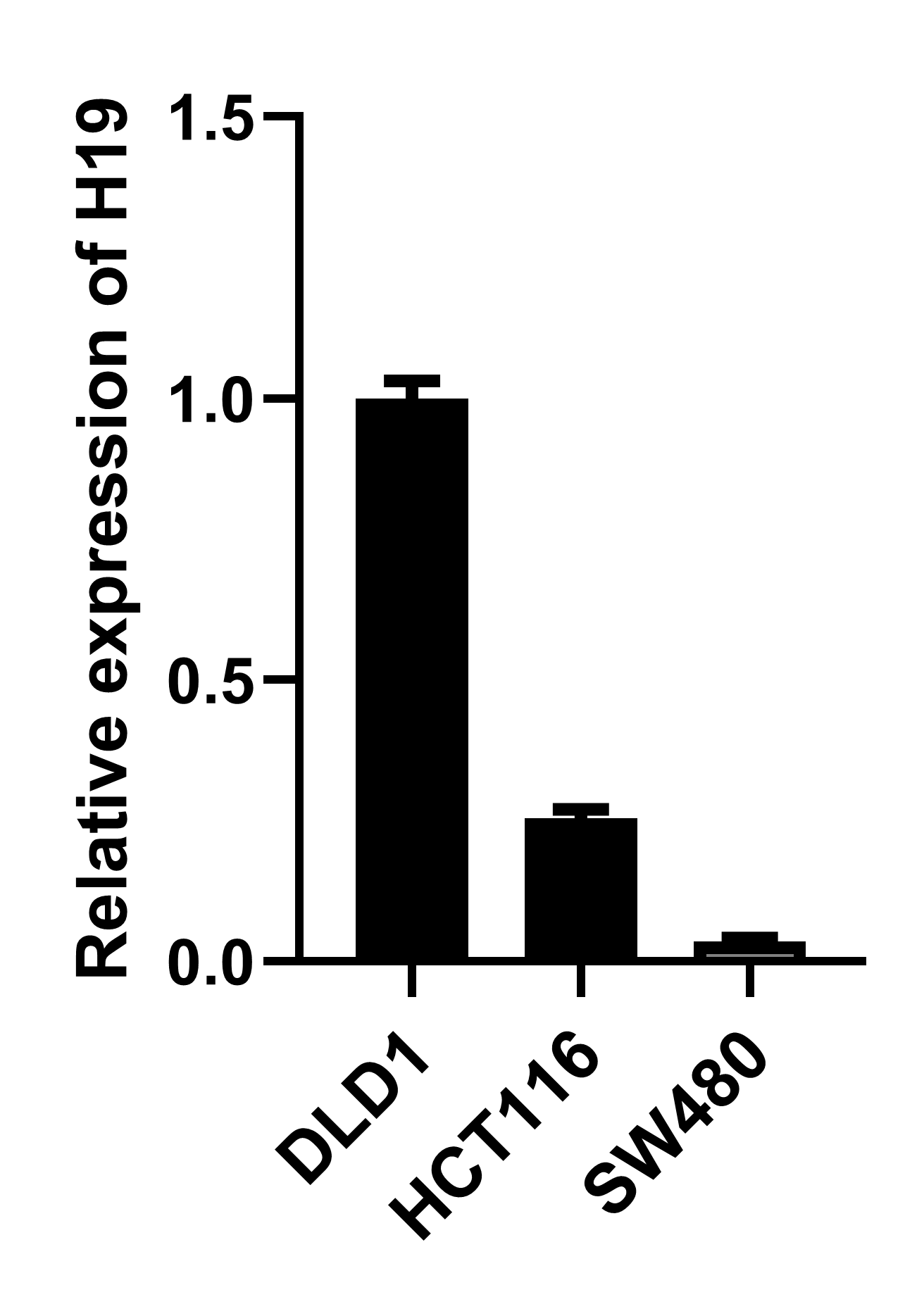

Supplement: Supplementary file 1 — Additional file 1. [file 13046_2020_1619_MOESM1_ESM.tif]

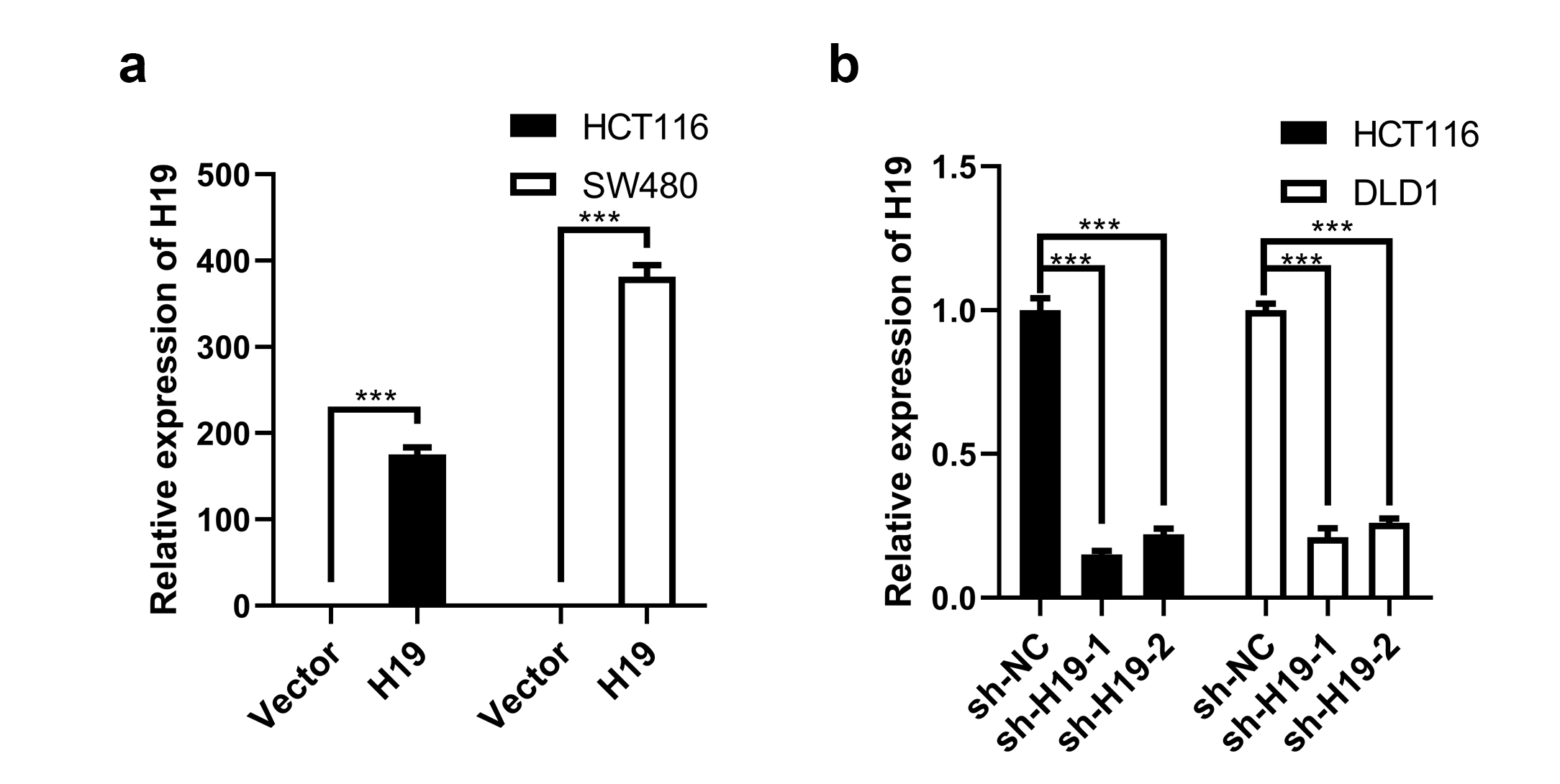

Supplement: Supplementary file 2 — Additional file 2. [file 13046_2020_1619_MOESM2_ESM.tif]

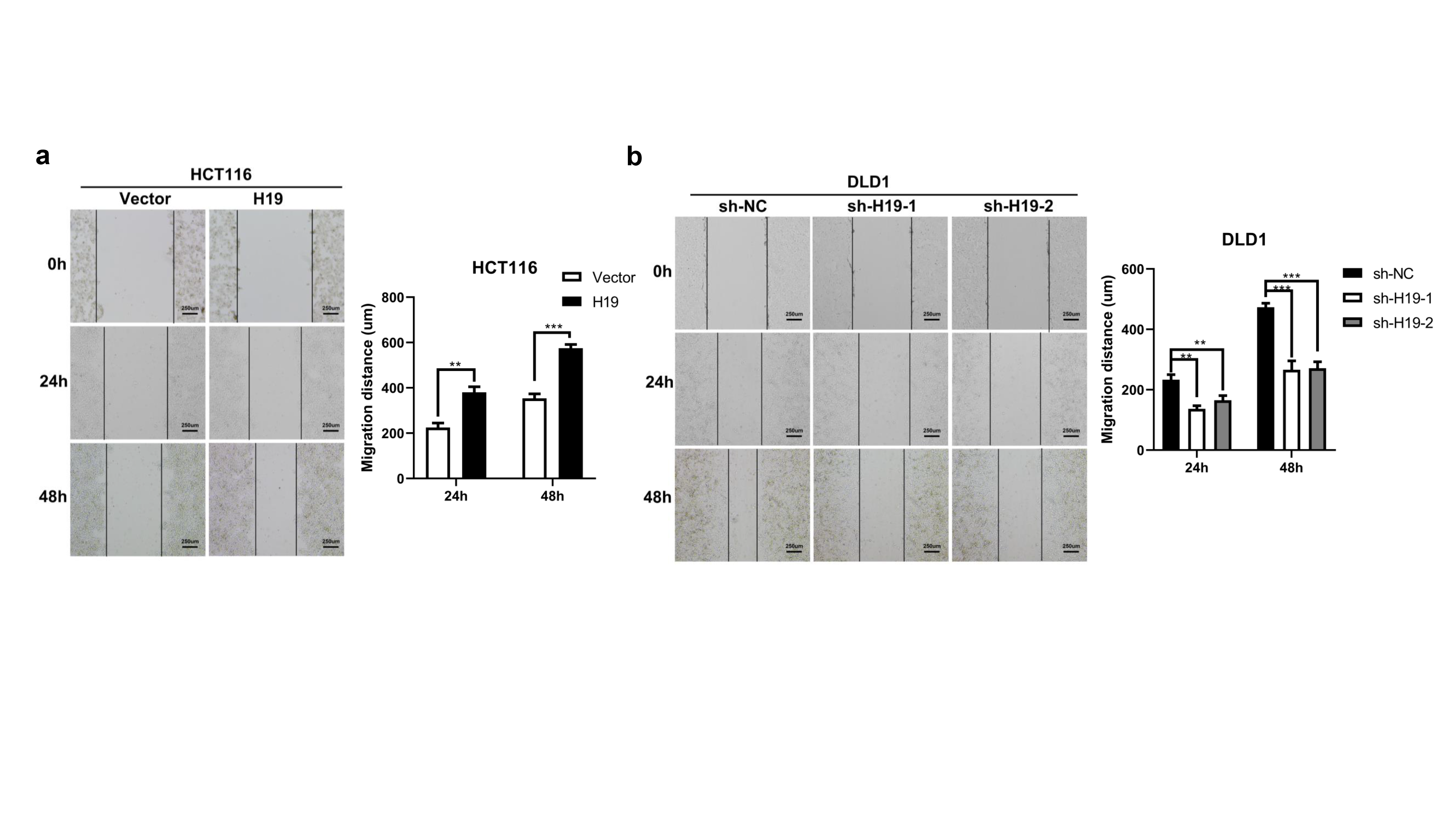

Supplement: Supplementary file 3 — Additional file 3. [file 13046_2020_1619_MOESM3_ESM.tif]

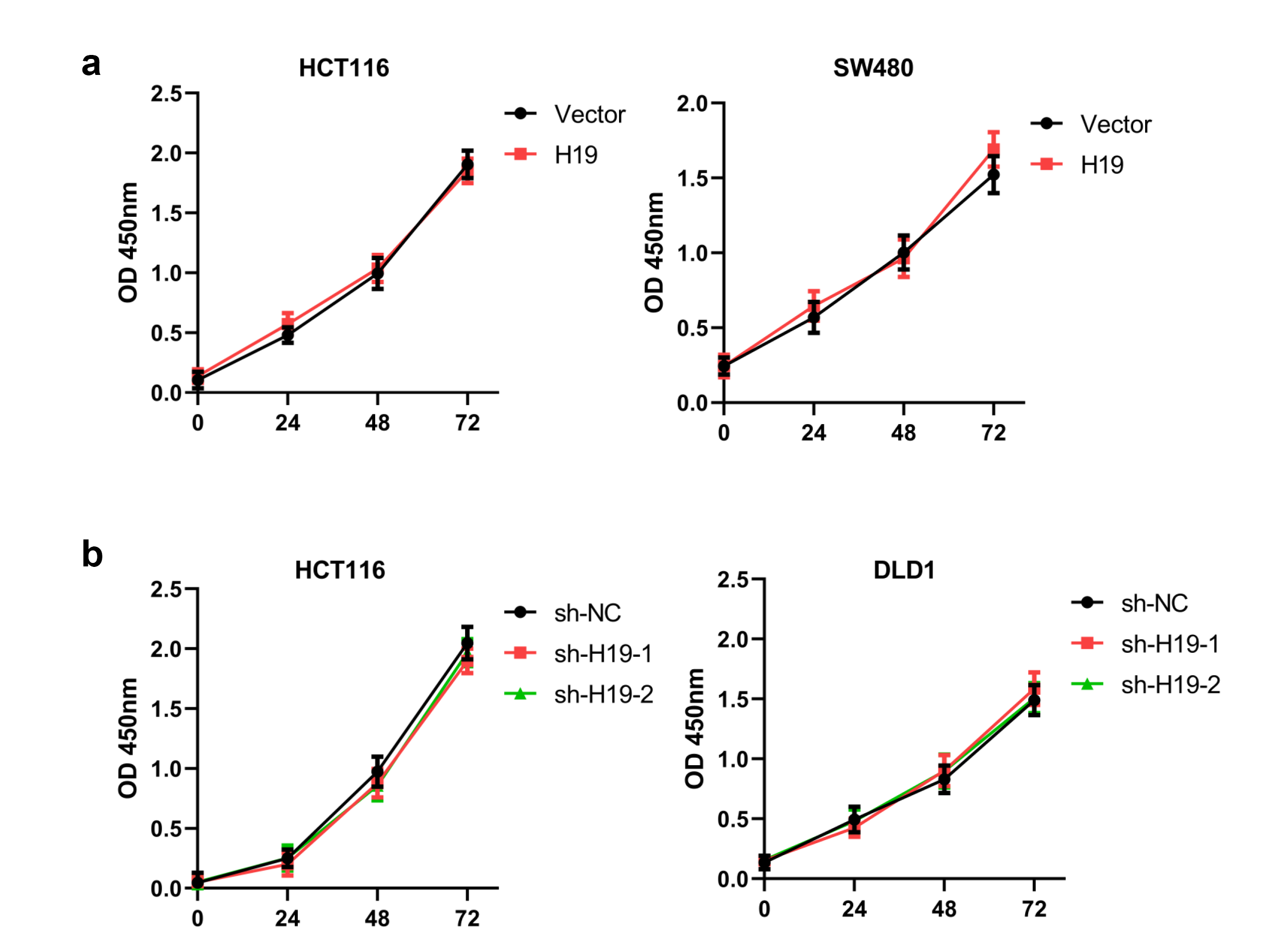

Supplement: Supplementary file 4 — Additional file 4. [file 13046_2020_1619_MOESM4_ESM.tif]

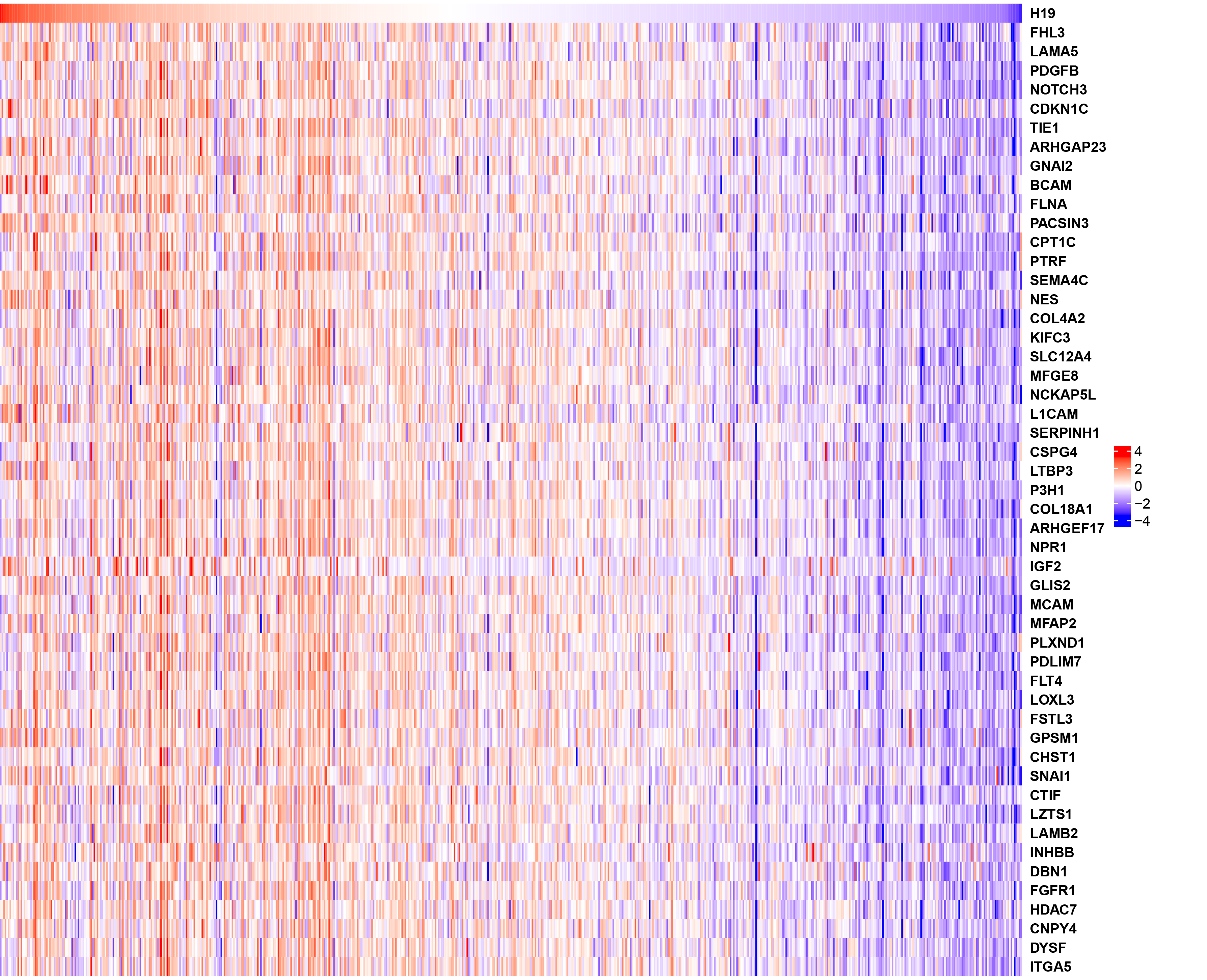

Supplement: Supplementary file 5 — Additional file 5. [file 13046_2020_1619_MOESM5_ESM.tif]

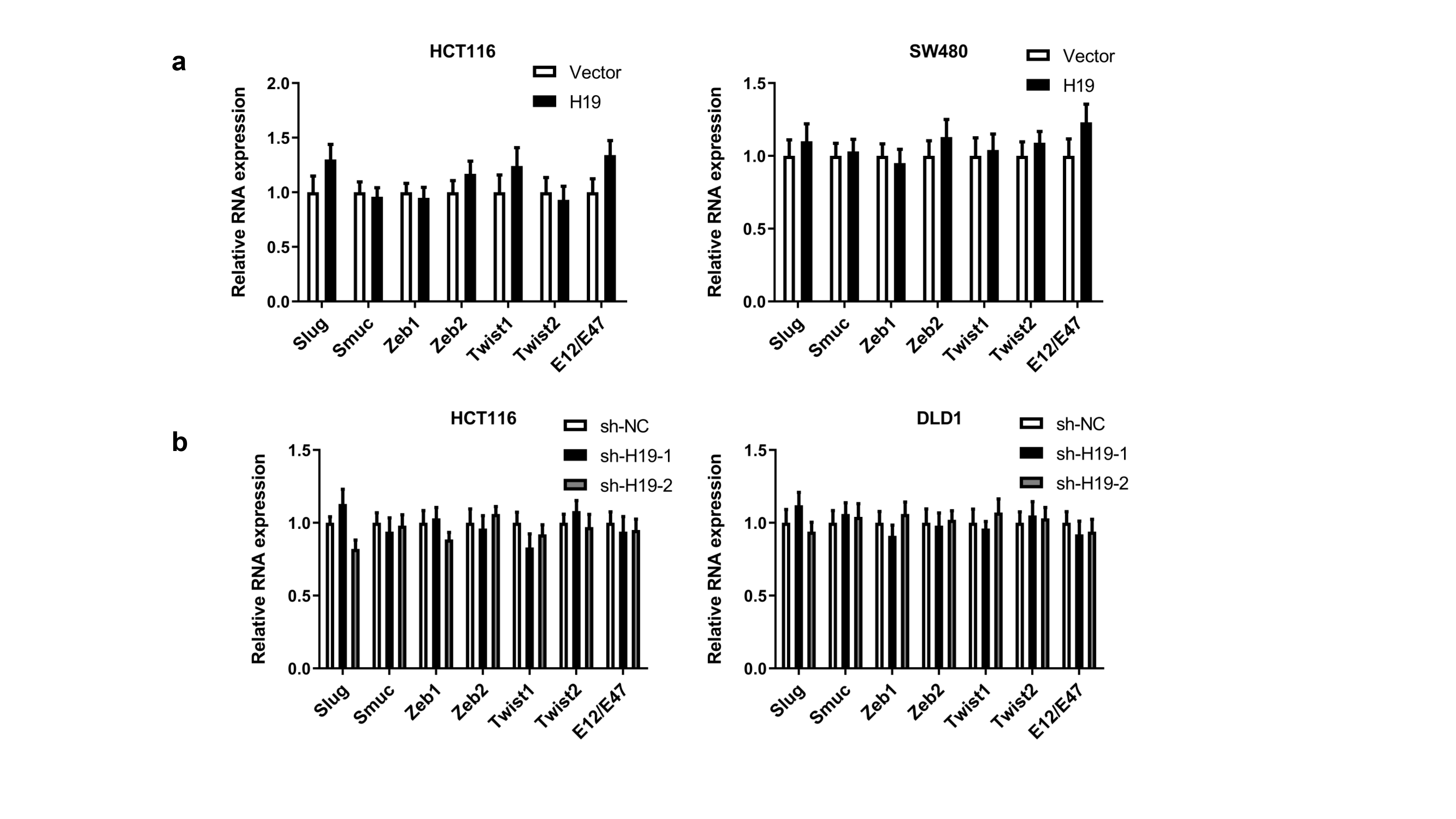

Supplement: Supplementary file 6 — Additional file 6. [file 13046_2020_1619_MOESM6_ESM.tif]

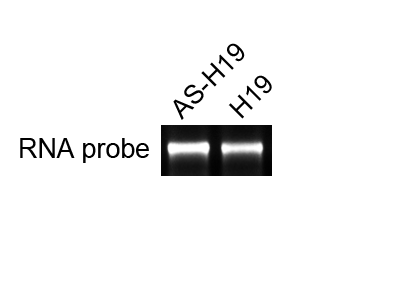

Supplement: Supplementary file 7 — Additional file 7. [file 13046_2020_1619_MOESM7_ESM.tif]

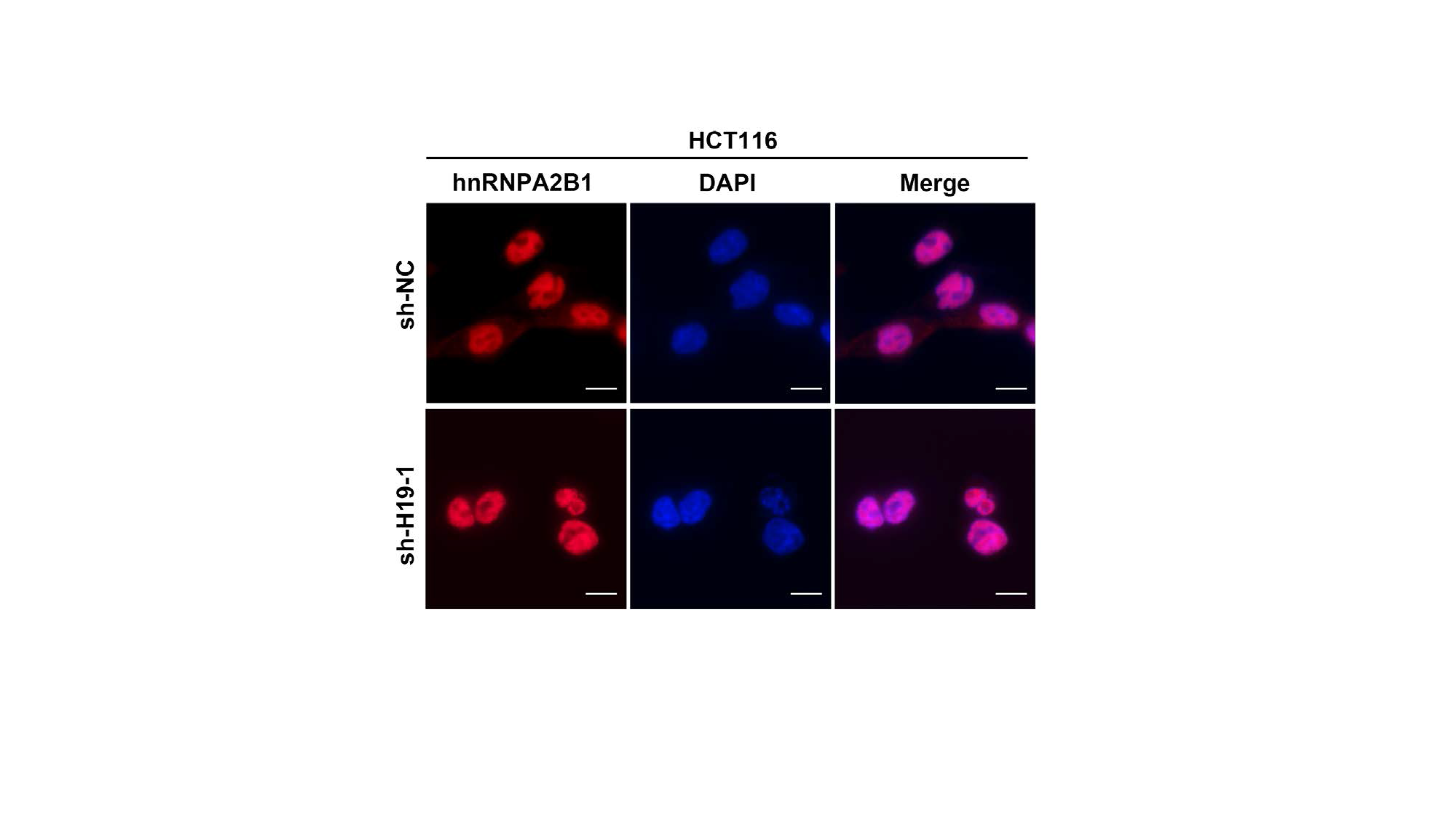

Supplement: Supplementary file 8 — Additional file 8. [file 13046_2020_1619_MOESM8_ESM.tif]

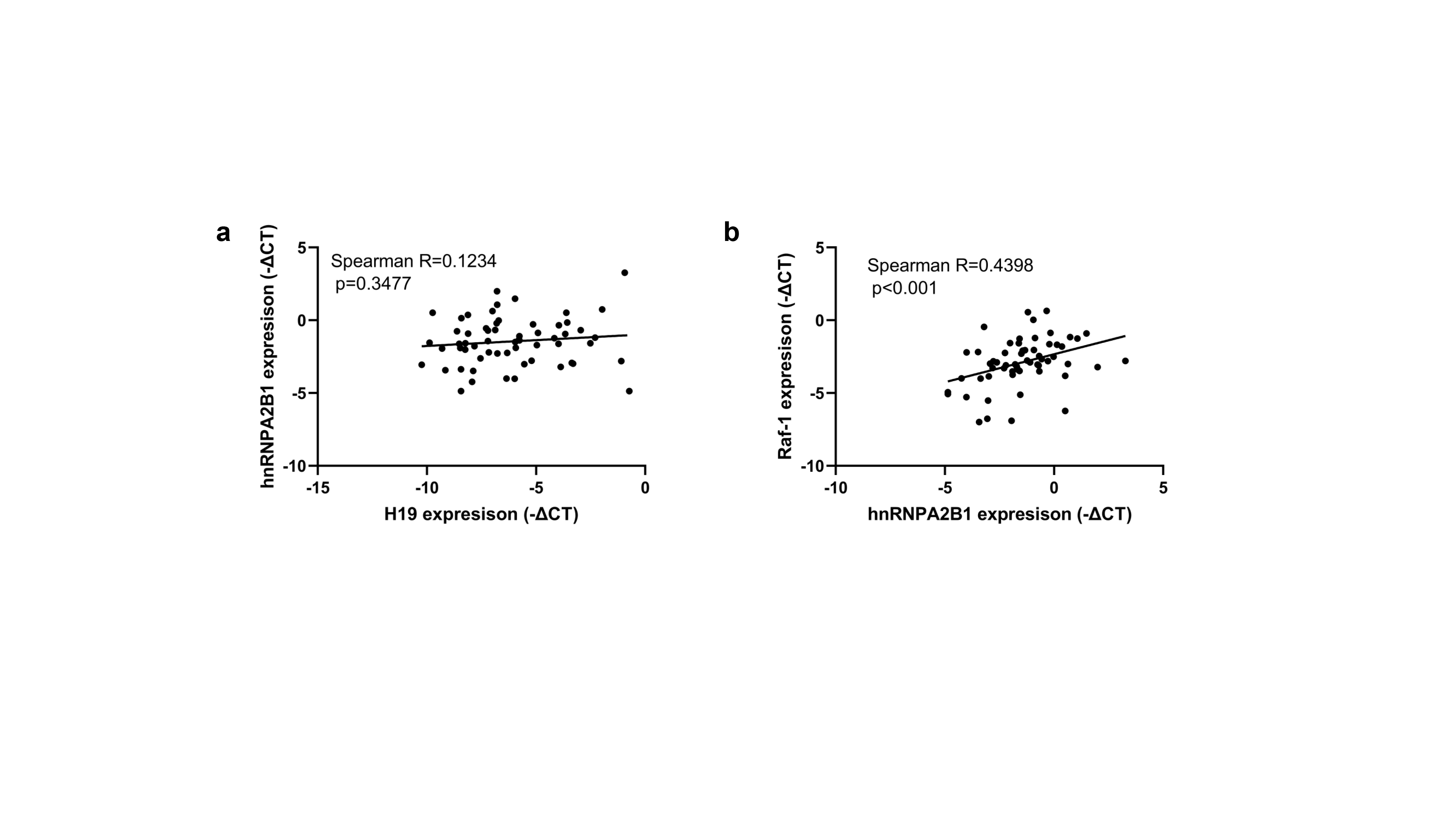

Supplement: Supplementary file 9 — Additional file 9. [file 13046_2020_1619_MOESM9_ESM.tif]

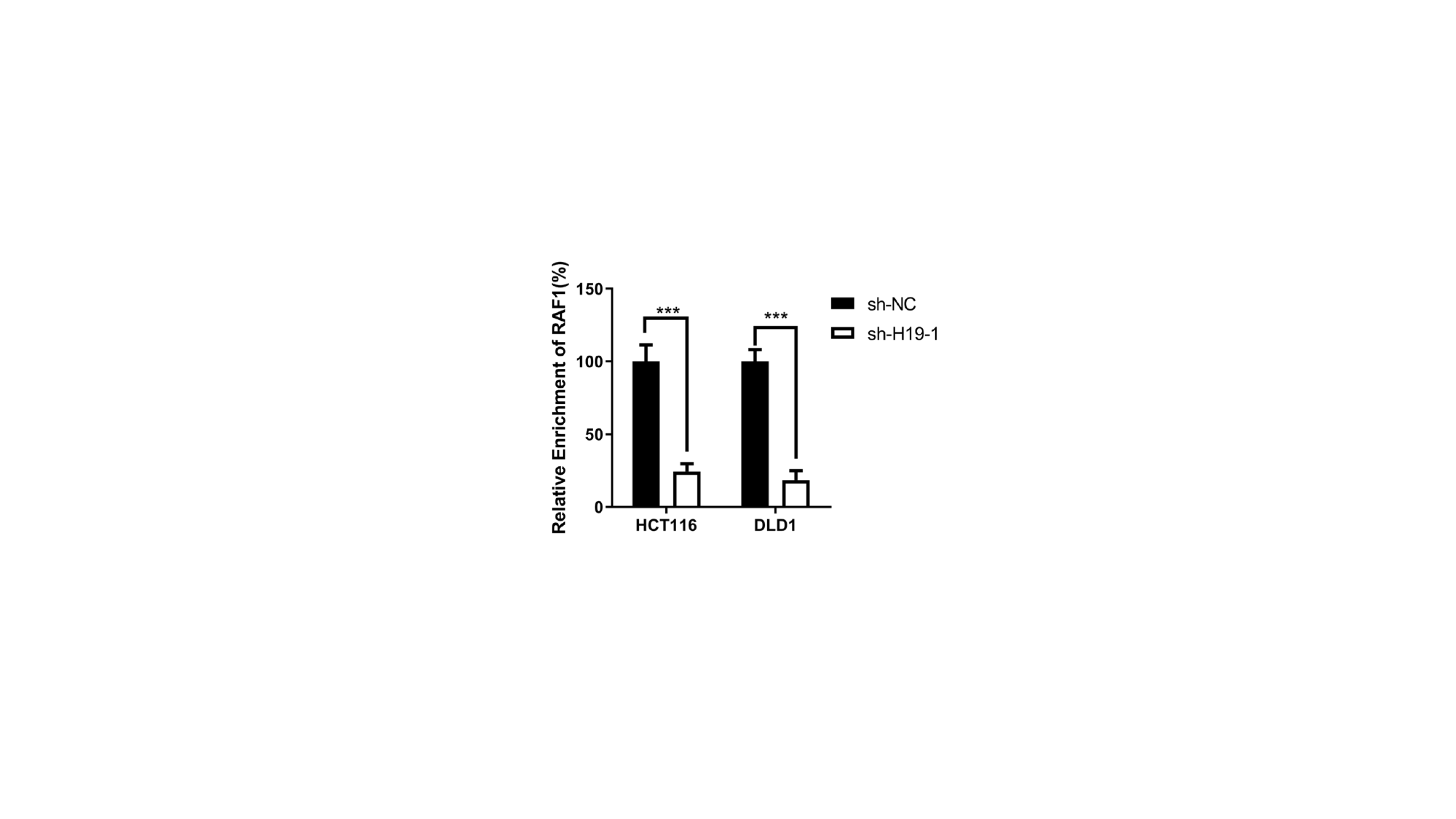

Supplement: Supplementary file 10 — Additional file 10. [file 13046_2020_1619_MOESM10_ESM.tif]
