# Supplementary material for: Long non-coding RNA H19 promotes colorectal cancer metastasis via binding to hnRNPA2B1
Source: J Exp Clin Cancer Res. 2020 Jul 23;39:141. doi: 10.1186/s13046-020-01619-6 (PMC7412843; doi:10.1186/s13046-020-01619-6)
Supplement: Supplementary file 11 — Additional file 11: Table S1. Target sequences of siRNA Table S2. Target sequences of shRNA Table S3. Sequences of primers [file 13046_2020_1619_MOESM11_ESM.docx]

Table. S1 Target sequences of siRNA

| Name | Target sequences |
| --- | --- |
| si-hnRNPA2B1-1 | GGAGAGTAGTTGAGCCAAA |
| si-hnRNPA2B1-2 | AGCTGTTTGTTGGCGGAAT |

Table. S2 Target sequences of shRNA

| Name | Target sequences |
| --- | --- |
| sh-H19-1 | GAGTTAGCAAAGGTGACATCT |
| sh-H19-2 | GCTCTGGAAGGTGAAGCTAGA |

Table. S3 Sequences of primers

| Name |  | Sequences |
| --- | --- | --- |
| GAPDH | Forward | 5'- ACAACTTTGGTATCGTGGAAGG-3' |
|  | Reverse | 5'-GCCATCACGCCACAGTTTC-3' |
| H19 | Forward | 5'_TGCTGCACTTTACAACCACTG-3' |
|  | Reverse | 5'-ATGGTGTCTTTGATGTTGGGC-3' |
| Snail | Forward | 5'-TCGGAAGCCTAACTACAGCGA-3' |
|  | Reverse | 5'-AGATGAGCATTGGCAGCGAG-3' |
| Slug | Forward | 5'-CGAACTGGACACACATACAGTG-3' |
|  | Reverse | 5'-CTGAGGATCTCTGGTTGTGGT-3' |
| Smuc | Forward | 5'-ACTGCGACAAGGAGTACACC-3' |
|  | Reverse | 5'-GAGTGCGTTTGCAGATGGG-3' |
| Zeb1 | Forward | 5'-GATGATGAATGCGAGTCAGATGC-3' |
|  | Reverse | 5'-ACAGCAGTGTCTTGTTGTTGT-3' |
| Zeb2 | Forward | 5'-GGAGACGAGTCCAGCTAGTGT-3' |
|  | Reverse | 5'-CCACTCCACCCTCCCTTATTTC-3' |
| Twist1 | Forward | 5'-GTCCGCAGTCTTACGAGGAG-3' |
|  | Reverse | 5'- GCTTGAGGGTCTGAATCTTGCT-3' |
| Twist2 | Forward | 5'-GGGAGTGAGCACATTAGCAA-3' |
|  | Reverse | 5'-GGGCATGAGTACCCTTAGGA-3' |
| E12/E47 | Forward | 5'-CCGACTCCTACAGTGGGCTA-3' |
|  | Reverse | 5'-CGCTGACGTGTTCTCCTCG-3' |
| hnRNPA2B1 | Forward | 5'-ATTGATGGGAGAGTAGTTGAGCC-3' |
|  | Reverse | 5'-AATTCCGCCAACAAACAGCTT-3' |
| Raf-1 | Forward | 5'-GGGAGCTTGGAAGACGATCAG-3' |
|  | Reverse | 5'-ACACGGATAGTGTTGCTTGTC-3' |
| A-Raf | Forward | 5'-CTAAATCAGGACTGCTGTGTGG-3' |
|  | Reverse | 5'-GACATCTTCAAGGACCTCGAC-3' |
| B-Raf | Forward | 5'-AATACACCAGCAAGCTAGATGC-3' |
|  | Reverse | 5'- AATCAGTTCCGTTCCCCAGAG-3' |
| β-actin | Forward | 5'- CATGTACGTTGCTATCCAGGC -3' |
|  | Reverse | 5'- CTCCTTAATGTCACGCACGAT -3' |
| U6 | Forward | 5’-GGAACGATACAGAGAAGATTAGC-3' |
|  | Reverse | 5’-TGGAACGCTTCACGAATTTGCG-3’ |
