# Supplementary material for: Long non-coding RNA H19 promotes colorectal cancer metastasis via binding to hnRNPA2B1
Source: J Exp Clin Cancer Res. 2020 Jul 23;39:141. doi: 10.1186/s13046-020-01619-6 (PMC7412843; doi:10.1186/s13046-020-01619-6)
Supplement: Supplementary file 13 — Additional file 13: Figure S1. The expression of H19 in different colorectal cancer cell lines detected by qRT-PCR. Figure S2. a The expression of H19 in HCT116 and SW480 cells after transfected with lv-H19 and control lv-Vector to construct stable H19 overexpression cell lines. b The expression of H19 in HCT116 and DLD1 cells after transfected with sh-NC, sh-H19-1 and sh-H19-2 to construct stable H19 knockdown cell lines. Student’s t-test. *P < 0.05, **P < 0.01, ***P < 0.001 Figure S3. a Wound healing assay in HCT116 H19 stable overexpression and control. The relative migration rate is calculated compared to the distance of 0h. b Wound healing assay in H19 knockdown and control DLD1 cells. Scales bars = 250um. Student’s t-test. *P < 0.05, **P < 0.01, ***P < 0.001 Figure S4. a CCK-8 assays of H19 overexpression or control cells. b CCK-8 assays of H19 knockdown or control cells. Student’s t-test. *P < 0.05, **P < 0.01, ***P < 0.001 Figure S5. Heatmap of the top 50 genes which H19 is most corelated with in TCGA database. Figure S6. a Effect of H19 overexpression on the mRNA level of EMT transcript factors in HCT116 and SW480 cells were measured by qRT-PCR. b Effect of H19 knockdown on the mRNA level of EMT transcript factors in HCT116 or DLD1 H19 cells were quantified by qRT-PCR. Figure S7. Agarose gel electrophoresis of RNA probe used for RNA pull-down assay. Figure S8. Immunofluorescence were performed to investigate the subcellular localization of hnRNPA2B1 in H19 depleted and control cells. Scales bars=10um. Figure S9. a Correlation analysis between H19 and hnRNPA2B1. b Correlation analysis between hnRNPA2B1 and Raf-1. Figure S10. RIP assay followed by qRT-PCR explored the enrichment of Raf-1 mRNA binding to hnRNPAA2B1 in H19 depleted and control cells. Student’s t-test. *P < 0.05, **P < 0.01, ***P < 0.001 [file 13046_2020_1619_MOESM13_ESM.docx]

**Fig. S1** The expression of H19 in different colorectal cancer cell lines detected by qRT-PCR.

**Fig. S2** **a** The expression of H19 in HCT116 and SW480 cells after transfected with lv-H19 and control lv-Vector to construct stable H19 overexpression cell lines. **b** The expression of H19 in HCT116 and DLD1 cells after transfected with sh-NC, sh-H19-1 and sh-H19-2 to construct stable H19 knockdown cell lines. Student’s t-test. *P < 0.05, **P < 0.01, ***P < 0.001

**Fig. S3 a** Wound healing assay in HCT116 H19 stable overexpression and control. The relative migration rate is calculated compared to the distance of 0h. **b** Wound healing assay in H19 knockdown and control DLD1 cells. Scales bars = 250um. Student’s t-test. *P < 0.05, **P < 0.01, ***P < 0.001

**Fig. S4 a** CCK-8 assays of H19 overexpression or control cells. **b** CCK-8 assays of H19 knockdown or control cells. Student’s t-test. *P < 0.05, **P < 0.01, ***P < 0.001

**Fig. S5** Heatmap of the top 50 genes which H19 is most corelated with in TCGA database.

**Fig. S6** **a** Effect of H19 overexpression on the mRNA level of EMT transcript factors in HCT116 and SW480 cells were measured by qRT-PCR. **b** Effect of H19 knockdown on the mRNA level of EMT transcript factors in HCT116 or DLD1 H19 cells were quantified by qRT-PCR.

**Fig. S7** Agarose gel electrophoresis of RNA probe used for RNA pull-down assay.

**Fig. S8** Immunofluorescence were performed to investigate the subcellular localization of hnRNPA2B1 in H19 depleted and control cells. Scales bars=10um.

**Fig. S9** **a** Correlation analysis between H19 and hnRNPA2B1. **b** Correlation analysis between hnRNPA2B1 and Raf-1.

**Fig. S10** RIP assay followed by qRT-PCR explored the enrichment of Raf-1 mRNA binding to hnRNPAA2B1 in H19 depleted and control cells. Student’s t-test. *P < 0.05, **P < 0.01, ***P < 0.001
